# TIP60 represses telomerase expression by inhibiting Sp1 binding to the *TERT* promoter

**DOI:** 10.1371/journal.ppat.1006681

**Published:** 2017-10-18

**Authors:** Deepa Rajagopalan, Amit Kumar Pandey, Magdalene Claire Xiuzhen, Kwok Kin Lee, Shainan Hora, Yanzhou Zhang, Boon Haow Chua, Hui Si Kwok, Shreshtha Sailesh Bhatia, Lih Wen Deng, Daniel G. Tenen, Dennis Kappei, Sudhakar Jha

**Affiliations:** 1 Cancer Science Institute of Singapore, National University of Singapore, Singapore; 2 Department of Biochemistry, Yong Loo Lin School of Medicine, National University of Singapore, Singapore; 3 Harvard Stem Cell Institute, Harvard Medical School, Boston, MA, United States of America; University of Wisconsin-Madison, UNITED STATES

## Abstract

HIV1-TAT interactive protein (TIP60) is a haploinsufficient tumor suppressor. However, the potential mechanisms endowing its tumor suppressor ability remain incompletely understood. It plays a vital role in virus-induced cancers where TIP60 down-regulates the expression of human papillomavirus (HPV) oncoprotein E6 which in turn destabilizes TIP60. This intrigued us to identify the role of TIP60, in the context of a viral infection, where it is targeted by oncoproteins. Through an array of molecular biology techniques such as Chromatin immunoprecipitation, expression analysis and mass spectrometry, we establish the hitherto unknown role of TIP60 in repressing the expression of the catalytic subunit of the human telomerase complex, TERT, a key driver for immortalization. TIP60 acetylates Sp1 at K639, thus inhibiting Sp1 binding to the *TERT* promoter. We identified that TIP60-mediated growth suppression of HPV-induced cervical cancer is mediated in part due to *TERT* repression through Sp1 acetylation. In summary, our study has identified a novel substrate for TIP60 catalytic activity and a unique repressive mechanism acting at the *TERT* promoter in virus-induced malignancies.

## Author summary

A common feature of all malignant cells is their ability of continued proliferation while avoiding replicative senescence. Tumors either activate expression of telomerase reverse transcriptase, *TERT* (in the case of ~ 85% of tumors) or use a recombination based alternative lengthening of telomeres (remaining 15% of tumors) to maintain their telomere length and thus ensure continued cell replication. Most viruses which cause tumors such as human papillomavirus (HPV) and Hepatitis B virus (HBV) also activate the expression of telomerase and result in malignancy. In this study, we have identified a novel cellular repressor of telomerase, TIP60. TIP60 which is targeted for degradation by HPV oncoprotein E6, acetylates Sp1, a positive regulator of *TERT*. We show that TIP60-mediated acetylation of Sp1, inhibits Sp1 function at the *TERT* promoter, thereby reducing *TERT* expression and thus the growth of HPV-positive cervical cancer cells, HeLa.

## Introduction

HIV-1 TAT Interactive protein (TIP60) is a lysine acetyltransferase and a member of the MYST (Moz, Ybf2/Sas3, Sas2 and TIP60) family of evolutionarily related histone acetyltransferases [1]. TIP60 can acetylate histone and non-histone proteins and is involved in a multitude of cellular functions like altering the chromatin structure, transcription and DNA damage repair response [2–4]. TIP60’s role as a *bona fide* tumor suppressor has also been documented [5]. In return, it is targeted for degradation by many viral oncoproteins like the human papillomavirus (HPV) oncoprotein E6 and adenoviral oncoproteins EIB55K and E4orf6 [6–8]. Infection by the oncogenic HPV is the leading cause of cervical cancer, the second most common cancer in women worldwide. High-risk HPV (HPV 16, HPV 18) is implicated in invasive carcinoma while low-risk HPV (HPV6, HPV8, HPV11) is responsible for genital warts and non-malignant lesions [9]. The mechanism of transformation of normal keratinocytes by HPV is dependent on two oncoproteins, E6 and E7, which interact with and destabilize the tumor suppressors p53 and pRb (Retinoblastoma protein) [10, 11]. E6 also destabilizes TIP60 by cooperating with an E3 cellular ubiquitin ligase, EDD1 [8], and TIP60 in turn represses the E6 promoter, thus maintaining a delicate balance of cellular and viral gene expression [7]. Interestingly, both high and low-risk HPV are capable of destabilizing TIP60 whereas only the high-risk E6 is capable of destabilizing p53. This suggests that TIP60 degradation is an essential and common mechanism in promoting viral gene expression [7, 11].

Telomeres consist of repetitive DNA at the ends of linear chromosomes and their degradation limits the replicative potential of cells [12, 13]. This is a consequence of progressive telomere shortening, occurring with every round of cell division due to the combined contributions of the end replication problem and active telomere processing [14, 15]. Like normal somatic cells, cancer cells would ultimately face telomere-induced replicative senescence but they (re-)activate one of two telomere maintenance pathways to bypass this fate: about 85% of all tumors reactivate telomerase, whereas the remaining 15% employ a recombination based maintenance mechanism, named Alternative Lengthening of Telomeres (ALT) [16, 17]. Telomerase is a reverse transcriptase that can add telomeric repeats *de novo*, thus counteracting telomere shortening. Its minimal core consists of a catalytic subunit, TERT, and an RNA template for reverse transcription, *TERC* [18]. *TERT* expression is regulated at the epigenetic, transcription and protein level by a multitude of cellular factors [19]. Additionally, many oncoviral proteins such as HPV E6 and HBV HBx are known to activate the expression of *TERT*, thus circumventing senescence and serving their purpose of cellular proliferation [20–23].

E6 collaborates with the transcription factor c-MYC to upregulate *TERT* in certain cervical cancer cell lines [24]. It also interacts with a cellular ubiquitin ligase E6AP to destabilize a repressor, NFX1-91, on the *TERT* promoter in a proteasome dependent manner [22, 25, 26]. The collaborative role of c-MYC and another transcription factor, Specificity Protein 1 (Sp1), to activate telomerase has also been reported [27]. This regulation occurs directly at the *TERT* promoter, which contains E boxes to enable binding of c-MYC and GC boxes that provide binding sites for Sp1 [28]. The dynamic relationship between E6 and TIP60, the reduction in the tumor size upon overexpression of TIP60 as well as reports of TIP60 being present at the *TERT* promoter [29], have led us to question if TIP60 can regulate *TERT* expression and if this regulation is dependent on E6. This study indeed identifies the hitherto unknown mechanism of *TERT* expression regulation by TIP60. We show that TIP60, in contrary to its role in acetylating histones leading to transcriptional activation, serves as a negative regulator of *TERT* expression by inhibition of Sp1 function. We show that TIP60 acetylates residue K639 on Sp1, which prevents Sp1 binding to the *TERT* promoter. Moreover, the biological significance of TERT repression by TIP60 is demonstrated by the ability of ectopically expressed TERT to rescue growth defects seen upon TIP60 overexpression in colony forming assays. Thus, this study identifies how TIP60 levels regulate *TERT* expression by limiting Sp1 binding to the *TERT* promoter.

## Results

### TIP60 regulates telomerase activity by altering mRNA expression of *TERT*

**Fig 1:**
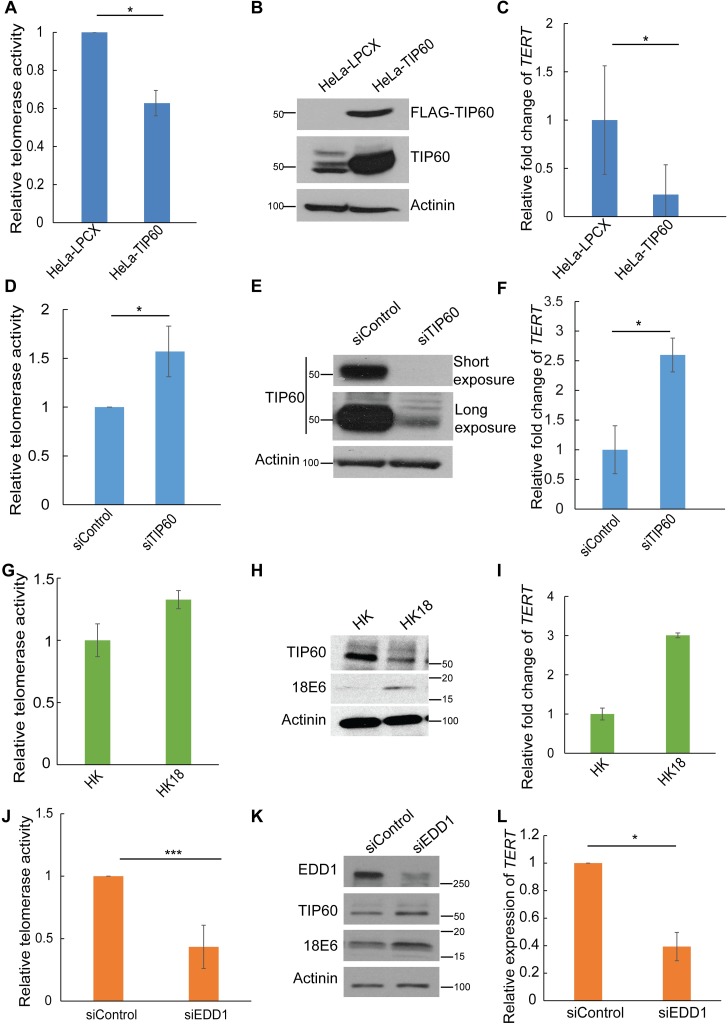
TIP60 regulates *TERT* expression and activity. **(A)** Telomerase activity was measured by qTRAP, using the whole cell lysates from HeLa-LPCX and HeLa-TIP60 cells. Heat inactivated samples served as negative control. Stable overexpression of Flag-tagged TIP60 in HeLa cells causes a decrease in the telomerase activity. **(B)** The stable cell lines generated were validated by western blotting using anti-Flag antibody. α-Actinin serves as a loading control to indicate equal protein amount used for qTRAP assay. **(C)** RNA was isolated from HeLa-LPCX and HeLa-TIP60 cells and real time PCR was performed to check for *TERT* expression. **(D)** TIP60 was transiently depleted in HeLa-TIP60 cells using siRNA and cells were harvested 72 h post transfection. Telomerase activity was measured. **(E)** Efficiency of TIP60 depletion was confirmed by western blotting. TIP60 was detected using an endogenous TIP60 antibody and α-Actinin serves as loading control. **(F)** Cellular RNA was isolated and analyzed by real time PCR after knockdown to determine *TERT* expression. mRNA expression was normalized to *GAPDH* and plotted as fold change. (**G**) Relative telomerase activity was measured for HK (human primary keratinocytes) and HK 18 (human keratinocytes expressing integrated HPV 18 genome) cells. (**H**) Detection of TIP60, 18 E6 and α-Actinin using antibodies for endogenous proteins in HK and HK18 cells. (**I**) RNA was isolated from HK and HK 18 and *TERT* expression was detected by qPCR. (**J**) Transient depletion of EDD1 in HeLa, the ubiquitin ligase known to destabilize TIP60 and measurement of relative telomerase activity. (**K**) Western blotting of the same samples harvested 72 h after siRNA transfection, with the indicated antibodies to detect endogenous levels of TIP60, EDD1 and E6. (**L**) qPCR to detect changes in *TERT* mRNA expression upon EDD1 depletion. Error bars reflect the standard error of mean (SEM) of 3 independent experiments and significance is represented as *, *P*<0.05.

Based on our previous finding of a feedback loop between TIP60 and HPV E6, and the phenotype of reduction in tumor size upon overexpression of TIP60 *in vivo* [7, 8], here we focused on elucidating the molecular mechanism of this tumor suppressor function of TIP60. Since HPV infection causes immortalization and TIP60 is destabilized by HPV oncogene E6, we investigated if TIP60 regulates *TERT* expression, a key factor involved in immortalization, in the context of the HeLa cervical cancer cell line that constitutively expresses the viral oncogenes E6 and E7 [30]. In order to investigate TIP60’s role in regulating telomerase activity, we generated HeLa-LPCX (vector) and HeLa-TIP60 overexpressing cell lines [8]. Stable overexpression of Flag-tagged TIP60 results in a decrease in telomerase activity as measured by the quantitative telomere repeat amplification protocol (qTRAP) (**Fig 1A and 1B)**, suggesting that the presence of TIP60 represses telomerase activity. The qTRAP assay serves as a readout for a functional telomerase complex and the associated enzymatic activity. Next, we sought to investigate if this repression was due to a change in the transcript levels of *TERT*. We measured mRNA levels of *TERT* in HeLa-LPCX and HeLa-TIP60 cells. In agreement with the changes in telomerase activity, TIP60-overexpressing cells show decreased *TERT* mRNA expression (**Fig 1C**). To validate this was specific to TIP60, we depleted TIP60 in HeLa-TIP60 cells by small interfering RNA (siRNA) transfection. Upon transient depletion of TIP60 **(Fig 1E)**, we observed an increase in both telomerase activity and *TERT* expression (**Fig 1D and 1F**). The siTIP60 siRNA is a commercially available pool of siRNAs which targets TIP60 and is designed to minimize off-target effects. To further rule out off-target effects of the siRNA, we used another siRNA (siTIP60A) to repeat these experiments in HeLa-TIP60 cells. Transient knockdown of TIP60 using siTIP60A also resulted in an increase in *TERT* mRNA expression and consequently an increase in relative telomerase activity **(S1A, S1B** and **S1C Fig)**. Consistent with our observations in HeLa-TIP60 cells, TIP60 depletion in the HeLa parental cells also increased *TERT* mRNA expression and activity (**S1D, S1E** and **S1F Fig**). For all the further experiments, the pool siRNA, siTIP60 has been used.

In order to study if this *TERT* repression is also observed in primary keratinocytes, which represent a relevant physiological system that bears similarities to early stages of tumorigenesis, we compared *TERT* expression and TIP60 levels between primary human keratinocytes (HK) and primary keratinocytes which have HPV 18 (HK 18) genome integrated. Consistent with our hypothesis and literature, TIP60 expression was reduced in HK 18 compared to HK cells (**Fig 1H**). This was also reflected in the increased expression of *TERT* as well as the increase in relative telomerase activity in HK 18 compared to HK cells (**Fig 1G and 1I**).

To ensure that these effects were not cell line-specific, we depleted TIP60 in CaSki-TIP60 cells generated using the same retroviral infection as described earlier for HeLa [8]. CaSki is also a cervical cancer line, but these cells constitutively express another high-risk E6 protein, 16E6. Depletion of TIP60 in CaSki-TIP60 cells also resulted in an increase both in telomerase activity (**S1G and S1H Fig**) and *TERT* mRNA levels (**S1I Fig)**. This data suggests that TIP60 inhibits telomerase activity by repressing *TERT* mRNA expression in cervical cancer cell lines and in primary keratinocytes compared to keratinocytes with integrated HPV 18 genome.

HeLa cervical cancer cells are dependent on E6 expression for circumventing senescence and regulating proliferation [30]. Apart from its critical role of degrading tumor suppressor p53 [11], E6 also activates telomerase [22], although the exact mechanism is complex and incompletely characterized. Additionally, the regulatory loop between E6 and TIP60 has already been established [7]. Thus it was imperative to study if the regulation of *TERT* by TIP60 is dependent on E6. In order to study this intricate feedback, HeLa cells were depleted transiently of both E6 and TIP60 using siRNA transfection and analyzed by western blotting using endogenous TIP60 and E6 antibodies (**S2A Fig**). In TIP60-depleted samples, the telomerase activity was found to be increased, consistent with our earlier results, and was rescued to control level in the E6 and TIP60 co-depleted samples (**S2B Fig**). This data suggests that the TIP60 repression of *TERT* is E6-dependent in cells expressing E6. However, this regulation of *TERT* by TIP60 is a mechanism that seems to be conserved in cervical cancer cells and is not restricted to only HPV positive cells. C-33A cells, cervical cancer cells without HPV infection, also showed an induction of *TERT* expression upon TIP60 knockdown **(S2C Fig)**. Furthermore, HPV E6 cooperates with a cellular ligase EDD1 to destabilize TIP60, thus regulating TIP60 levels [8]. In order to study the TIP60-E6-TERT dynamic, and the effect of a physiological increase in TIP60 level in HeLa cells, we transiently depleted EDD1 using siRNA (**Fig 1K**) and observed a decrease in *TERT* expression and telomerase activity consistent with the stabilization of TIP60 (**Fig 1J and 1L**).

### *TERT* promoter is regulated by TIP60 through the transcription factor Sp1

**Fig 2:**
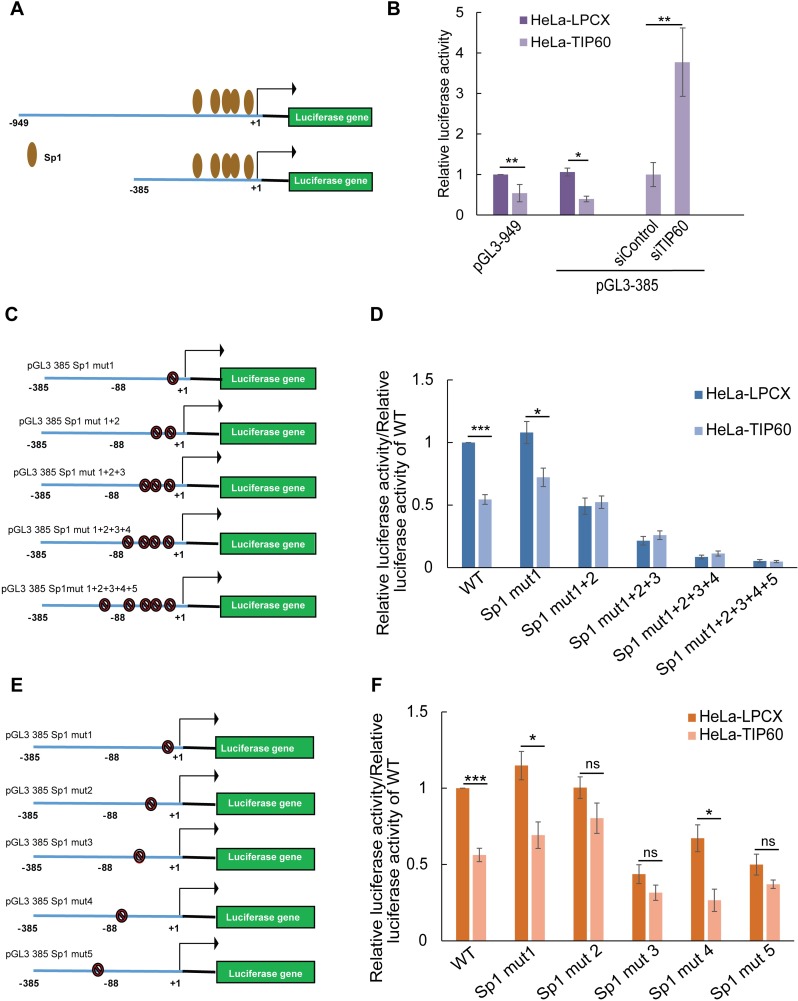
TIP60 regulates the *TERT* promoter. **(A)** Schematic representation of various *TERT* promoter fragments driving the expression of luciferase gene. Also seen are the known binding sites of the transcription factor, Sp1. **(B)** HeLa-LPCX (vector), HeLa-TIP60 and HeLa-TIP60 sicontrol/ siTIP60 cells were transiently transfected with luciferase constructs and the cell lysates were processed for luciferase assay after 24 h. **(C)** Schematic representation of the mutations generated on the *TERT* promoter of the 5 Sp1 binding sites in the pGL3-385 (WT) luciferase construct. **(D)** Reporter gene assay with the mutant constructs transiently transfected in HeLa-LPCX and HeLa-TIP60 cells. **(E)** Schematic of the individual Sp1 site mutations in the pGL3-385 construct. **(F)** Mutant constructs were transfected into HeLa-LPCX and HeLa-TIP60 cells and luciferase reading was assayed. Relative luciferase activity was plotted as a ratio of firefly to Renilla luciferase activity. For the mutants, luciferase reading was plotted normalized to the pGL3-385 (WT) construct. Error bars reflect the standard error of mean (SEM) of at-least 3 independent experiments and significance is represented as ***, *P*<0.001, **, *P*<0.01, *, *P*<0.05.

One possible explanation for *TERT* transcriptional repression is the regulation at the level of the *TERT* promoter. To test this, we used different constructs of the *TERT* promoter driving the expression of luciferase in a reporter gene assay (**Fig 2A)**. We used constructs of different length, covering either the full-length promoter sequence (-949 bp to +40 bp) or a shorter version only covering the proximal promoter (-385 bp to +40 bp) [31]. The two constructs were transiently transfected into HeLa-LPCX and HeLa-TIP60 cells and luciferase activity was measured. Compared to HeLa-LPCX cells, the luciferase activity was significantly reduced in HeLa-TIP60 cells, suggesting that TIP60 negatively regulates the *TERT* promoter (**Fig 2B**). We observed that the TIP60 repression of luciferase activity occurred in both the promoter constructs. In order to eliminate any cell line specific effect that may arise on comparison of HeLa-LPCX and HeLa-TIP60 cells and also to ascertain specificity of TIP60 to *TERT* promoter regulation, we transiently depleted TIP60 in HeLa-TIP60 cells. 24 h prior to harvesting, these cells were transfected with pGL3-385 reporter construct and luciferase activity was measured. Consistent with our previous findings, depletion of TIP60 resulted in an increase in *TERT* expression (**S3A and S3B Fig**) as well as an increase in the luciferase activity (**Fig 2B**). As previously reported, the first 181 bp of the *TERT* promoter contains 5 Sp1 binding sites and a single c-MYC binding site. MAZ, another transcription factor, recognizes and binds GA boxes on promoters of target genes and regulates expression of cellular genes such as c-MYC and Sp1. In order to study if MAZ, Sp1 or MYC play a role in TIP60 mediated *TERT* repression, first, TIP60 and MAZ were depleted transiently in HeLa cells. There was a decrease in *MAZ* expression upon TIP60 depletion. However, it was observed that *TERT* increases both upon depletion of TIP60 alone and also with co-depletion of TIP60 and MAZ (**S5E and S5F Fig**), thereby suggesting that MAZ may not be involved in this process.

Sp1 is known to be involved in the regulation of *TERT* in collaboration with c-MYC by binding to the GC boxes on the *TERT* promoter [27] (**Fig 2A, S5A Fig**), thus making both Sp1 and c-MYC plausible candidates for TIP60 mediated repression. To test if TIP60 regulates *TERT* promoter through Sp1 or c-MYC, we sequentially mutated the 5 Sp1 binding sites as well as the single c-MYC binding site using site-directed mutagenesis in the pGL3-385 construct of *TERT* promoter and assessed luciferase activity in HeLa-LPCX and HeLa-TIP60 cells (**Fig 2C, S5B Fig**) [27]. While mutating the c-MYC binding site did not affect the TIP60-mediated repression, TIP60-overexpressing cells were no longer able to repress the *TERT* promoter upon mutation of two or more Sp1 binding sites (**Fig 2D and S5C Fig**). There was a progressive decrease in the luciferase activity with each consecutive mutation of Sp1 binding site consistent with earlier studies (**Fig 2D**) [27]. To identify the Sp1 binding sites that are affected by TIP60, we also mutated each of the 5 Sp1 binding sites individually using site-directed mutagenesis (**Fig 2E**). Upon transient transfection of the mutants in HeLa-LPCX and HeLa-TIP60 cells, we observed that mutations of binding sites 1 and 4 did not significantly rescue the TIP60-mediated *TERT* promoter repression. However, mutation of binding sites 2, 3 and 5 partially abolished the repressive effect of TIP60 (**Fig 2F**). This data suggests that TIP60 represses the *TERT* promoter by inhibiting Sp1.

### TIP60 inhibits Sp1 occupancy at the *TERT* promoter

**Fig 3:**
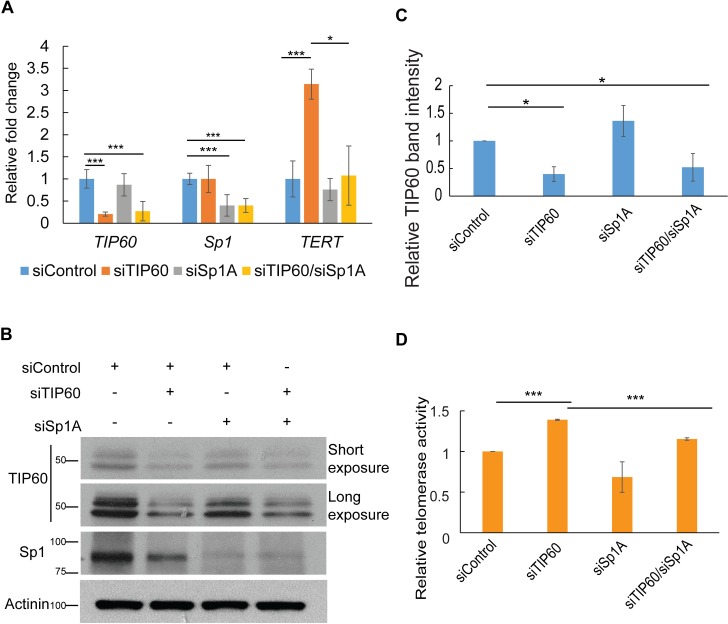
TIP60 inhibits Sp1 function. **(A, B)** TIP60 and Sp1 were depleted in HeLa cells using siRNA transfection. Cells were harvested 72 h post transfection, RNA and protein was isolated to use for real time PCR and western blotting analysis. Real-time PCR analysis was used to study *TERT* expression upon TIP60 and Sp1 knockdown. All mRNA expression data was normalized to *GAPDH* and plotted as fold change. Endogenous TIP60 and Sp1 were detected using anti-TIP60 and anti-Sp1 antibodies respectively. All the bands observed when probed with anti-TIP60 correspond to TIP60 protein since they are specifically reduced upon depletion of TIP60. α-Actinin serves as the loading control for the western blot. (**C**) Quantification of TIP60 bands using Image J software, from three independent experiments has also been depicted. **(D)** Relative telomerase activity was measured by qTRAP in the same set of samples. Error bars reflect the standard error of mean (SEM) of 3 independent experiments and significance is represented as ***, *P*<0.001, *, *P*<0.05.

To determine the exact role of Sp1 in TIP60-mediated *TERT* repression, we first co-depleted TIP60 and Sp1 in HeLa cells using siRNA transfection. The knockdown efficiencies of both Sp1 and TIP60 were greater than 70% both at mRNA and protein levels (**Fig 3A, 3B and 3C**). The increase in telomerase activity and *TERT* expression observed upon TIP60 knockdown was partially rescued by co-depletion of TIP60 and Sp1, suggesting that Sp1 acts downstream of TIP60 (**Fig 3A and 3D)**. That *TERT* expression as well as telomerase activity were reduced upon depletion of Sp1 alone, although to a small extent, is consistent with the findings from other groups (**Fig 3A and 3D**) [32]. In order to rule out off-target effects of siRNA mediated knockdown, we used another siRNA for depleting Sp1, siSp1 B, and observed a similar rescue in *TERT* expression upon co-depletion of TIP60 and Sp1 (**S4A and S4B Fig**).

**Fig 4:**
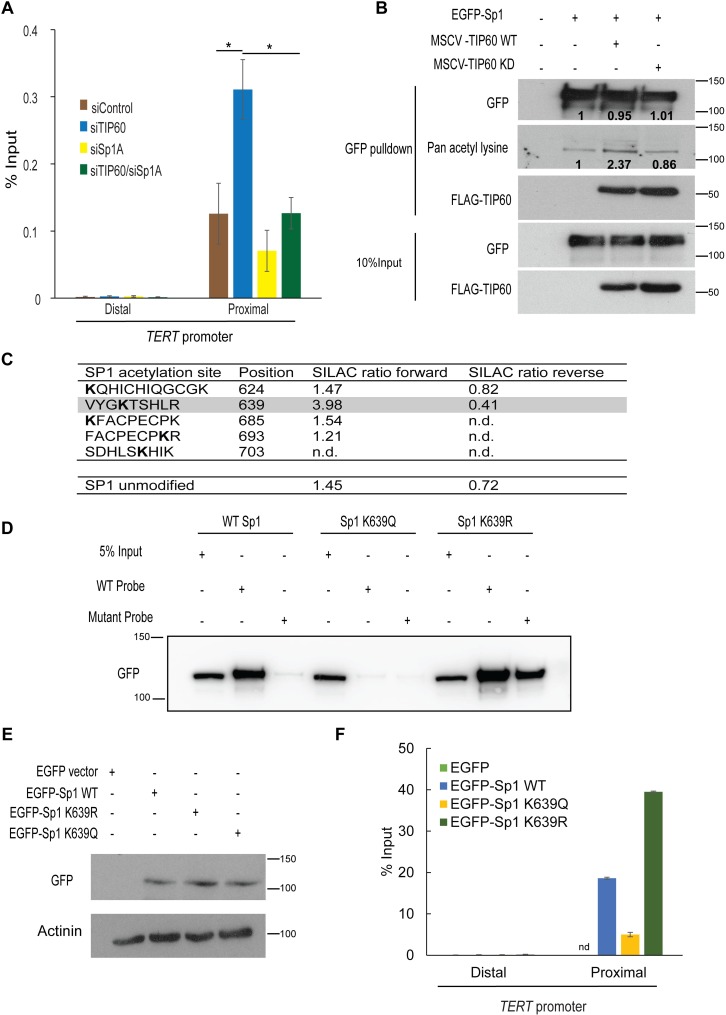
TIP60 acetylates Sp1 and represses its occupancy at the *TERT* promoter. **(A)** Chromatin immunoprecipitation (ChIP) of Sp1 was performed in HeLa cells depleted of either TIP60 or Sp1 individually or together. Cells were fixed with formaldehyde 72 h post transfection and used for ChIP. An upstream element (distal region) on the *TERT* promoter served as a negative control for Sp1 binding. Error bars reflect the standard error of mean (SEM) of three independent experiments and significance is represented as *, *P*<0.05. **(B)** 1.5 μg of EGFP-Sp1 plasmid was transiently transfected with 1.5 μg of wild-type and catalytically inactive TIP60 (TIP60WT and TIP60KD) into 293T cells and GFP pull-down was performed 48 h post transfection to investigate acetylation of Sp1. The cells were treated with HDAC inhibitors for 5 h prior to harvesting. Sp1 and TIP60 were detected using anti-GFP and anti-Flag antibody respectively. Pan-acetyl lysine antibody was used to detect acetylation on Sp1. Quantification of the bands was performed using Image J software. GFP pulldown was quantified after normalizing to the GFP input in each lane and the pan-acetyl lysine was calculated after normalizing to each individual GFP pulldown. (**C**) List of acetylated lysines identified on Sp1. SILAC ratios of individual modified peptides are compared to the SILAC ratio of the overall, unmodified Sp1 protein. The VYGKTSHLR peptide modified at position 639 with clearly distinct SILAC ratios is highlighted. (**D**) WT Sp1 and mutant constructs (Sp1K639R and Sp1K639Q) were transiently transfected in HeLa cells and harvested after 33 h. 100 μg of protein was used for the DNA pull-down and the bound Sp1 was detected with anti-GFP antibody. (**E**) Sp1 constructs were transfected in HeLa cells and 24 h post transfection, cells were harvested and detection of Sp1 variants was performed using GFP antibody. (**F**) ChIP of GFP-tagged Sp1 variants in HeLa was performed after transfection of the Sp1 constructs and crosslinking using formaldehyde. n.d refers to the peptides that were identified but not quantified in the experiment.

To test whether TIP60 regulates *TERT* expression by controlling Sp1 binding to the *TERT* promoter, we performed chromatin immunoprecipitations (ChIP) for Sp1 under TIP60-depleted conditions. Consistent with earlier findings, Sp1 was enriched on the *TERT* promoter as compared to an upstream element [32] (**S5D Fig, Fig 4A)**. Upon depletion of TIP60, Sp1 occupancy at the *TERT* promoter was increased, whereas co-depletion of TIP60 and Sp1 showed enrichment similar to control levels (**Fig 4A**). Together, these data suggest that TIP60 indeed represses *TERT* by inhibiting Sp1 binding to the *TERT* promoter and removing its effect as a transcriptional activator. We next sought to identify if TIP60 and Sp1 can associate with each other. Upon pulldown of endogenous TIP60, we identified interaction with endogenous Sp1 (**S6A Fig**). Since both Sp1 and TIP60 are nuclear proteins, we also repeated the IP in the presence of DNAse, and observed a higher enrichment of both the proteins in this fraction as well as the interaction between the two proteins (**S6A Fig**), suggesting that indeed TIP60 and Sp1 proteins were found to be associated with each other in HeLa cells.

### Acetylation of Sp1 on lysine 639 by TIP60 regulates Sp1’s DNA binding ability

As TIP60 is an acetyltransferase that can acetylate histone and non-histone proteins and was found to interact with Sp1, we next investigated whether such a post-translational modification was putatively regulating Sp1 in this context. To test this, we performed pull-down experiments in 293T cells due to their high transient transfection efficiency and robust expression of exogenous protein. 293T cells were transiently transfected with EGFP-Sp1 either alone or with wild-type (MSCV-TIP60WT) or catalytically inactive form of TIP60 (MSCV-TIP60KD). Sp1 was pulled down using GFP TRAP A beads and its acetylation status was determined using a pan-acetyl antibody. We observed that overexpression of wild-type TIP60 increased the acetylation status of Sp1 whereas acetylation levels were unaltered upon overexpression of the catalytically inactive TIP60 **(Fig 4B)**, thereby confirming that Sp1 is indeed a substrate for TIP60’s acetyltransferase activity. In order to identify the lysine residue(s) on Sp1 that are specifically acetylated by TIP60, we performed the GFP pull-down experiments in SILAC (Stable Isotope Labeling with Amino Acids in Cell Culture) labeled cells and analyzed the Sp1 acetylation status between cells overexpressing either TIP60WT or TIP60KD quantitatively by mass spectrometry **(S6B Fig)**. First we ensured that incorporation rate of the labeled amino acids in our input samples was satisfactory (**S6C Fig**). Here, specific acetylation sites displayed a differential SILAC ratio, whereas non-TIP60 dependent acetylation sites should have SILAC ratios of about 1:1. We identified 5 acetylated lysines on Sp1 (**Figs 4C and S6B**) of which we also identified a previously known residue K703, a substrate of CBP/p300 acetyltransferase activity [33]. The other four acetylation sites on Sp1 have not been previously reported in other studies. The acetylation spectra for these individual peptides was observed, confirming their existence in our system of study (**S7A, S7B and S7C Fig**). While most acetylation sites have SILAC ratios similar to the total Sp1 protein, the K639 acetylation site has SILAC ratios indicating specific acetylation by TIP60 (**Fig 4C**). Interestingly, the K639 residue is part of the first of three zinc finger domains which are involved in Sp1 DNA binding (**S6C Fig**) [34]. This led us to speculate that acetylation of Sp1 residue K639 by TIP60 might inhibit the occupancy of Sp1 on the *TERT* promoter by interfering with Sp1’s DNA binding ability. In order to test this hypothesis, using site-directed mutagenesis, we generated two mutations of Sp1, one which was non-acetylable (K639R) and another that mimicked constitutive acetylation (K639Q). We then performed *in vitro* reconstitution DNA pull-downs with wild-type Sp1 as well as the two mutants. We transfected wild-type Sp1 and the two mutants (Sp1 K639R and Sp1 K639Q) in HeLa cells and performed pull-down experiments using probes with either wild-type Sp1 binding sequence or the mutated Sp1 binding site sequence. We observed that wild-type Sp1 binds to the wild-type DNA probe but not to the probe containing mutated Sp1 binding site, demonstrating the specificity of the experiment. Strikingly, the mutant that mimics constitutive acetylation, Sp1 K639Q, could not bind to either the wild-type or mutant DNA. In contrast, Sp1 K639R, non-acetylable mimetic was found to bind non-specifically to both the wild-type and mutant DNA sequences (**Fig 4D**). To test if this binding behaviors were recapitulated *in vivo*, we transiently transfected these GFP tagged Sp1 constructs into HeLa cells (**Fig 4E**) and performed ChIP with GFP antibody to selectively pulldown only the exogenous Sp1 proteins. Consistent with the DNA pulldown results in **Fig 4D**, wild-type Sp1 protein was found to be enriched at the *TERT* promoter compared to the distal region (**Fig 4F**). Similar results were also obtained with Sp1 K639R mutant whereas the SP1 K639Q mutant was less enriched at the promoter region compared to both Sp1 WT and Sp1 K639R. This suggests that TIP60-mediated acetylation on Sp1 inhibits its DNA binding ability to the Sp1 binding sites found in the *TERT* promoter. This data shows that Sp1 is an acetylation target of TIP60 and that indeed acetylation at K639 inhibits its DNA binding function, thus providing a likely mechanism for TIP60-dependent transcriptional control of *TERT*.

### TIP60-dependent growth inhibition is due to repression of *TERT* expression

HeLa-TIP60 cells showed a significant reduction in colony formation ability *in vitro* and tumor formation *in vivo*. We questioned if this tumor suppressor role of TIP60 was dependent on repression of the *TERT* promoter and telomerase activity. Therefore in order to address the biological significance of TIP60-mediated *TERT* repression, we transiently transfected HeLa-LPCX and HeLa-TIP60 cells with either pBABE (vector) or pBABE-TERT. In this plasmid, *TERT* expression is not under the control of its endogenous promoter and could thus serve as a useful system to study if TIP60-mediated growth repression, is in part, facilitated by *TERT* downregulation and if it can be rescued by expression of exogenous *TERT*. 24 h post transfection, RNA was isolated from the same set of cells which were used in the colony formation assay (CFA) to study *TERT* expression and we observed a significant overexpression of *TERT* in both HeLa-LPCX and HeLa-TIP60 **(S8A Fig)**. There was also a decrease in *TERT* expression in HeLa-TIP60-pBABE as compared to HeLa-LPCX-pBABE, consistent with our earlier findings **(S8B Fig).**

**Fig 5:**
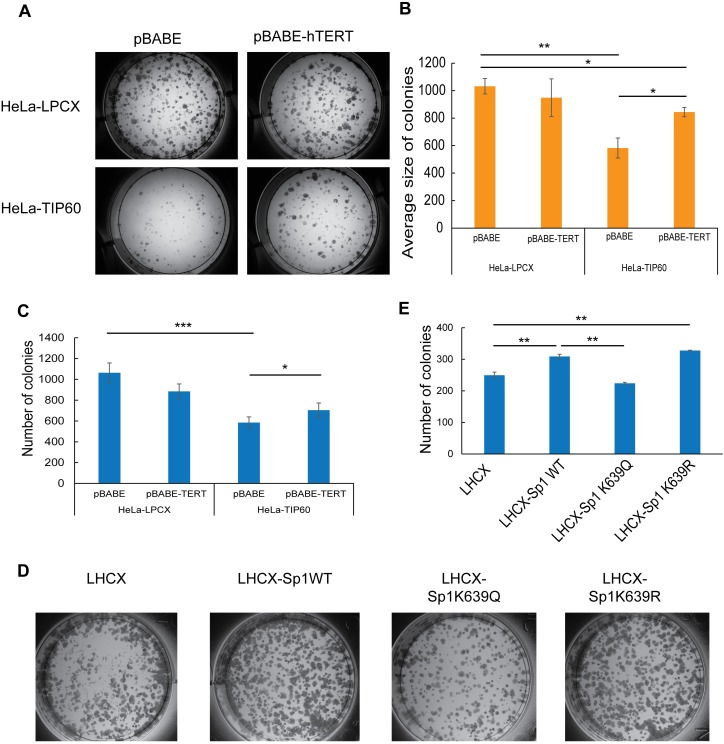
TIP60-mediated growth defects can be rescued by *TERT* overexpression and are dependent on Sp1 acetylation. (**A**) Representative image from Colony Formation Assay (CFA) where HeLa-LPCX and HeLa-TIP60 cells transiently transfected with pBABE (vector) or pBABE-TERT were seeded at a low density (2×10^3^ cells). The cells were allowed to grow for 11 days before fixing and staining with crystal violet. (**B, C**) Bar graph shows the quantification performed using Image J software for average size and number of colonies respectively. The overexpression of TIP60 reduces average colony size, which is rescued upon overexpression of *TERT* in HeLa-TIP60. (**D**) Sp1 plasmids in LHCX vector backbone were transfected into HeLa cells. Twenty four hours post transfection, 2500 cells were seeded for colony formation assay and representative images are shown. 11 days after, they were fixed and stained with crystal violet and (**E**) number of colonies was quantified using Image J software. Error bars reflect the standard error of mean (SEM) and significance is represented as *, *P*<0.05, **, *P*<0.01, ***, *P*<0.001.

2×10^3^ cells were seeded for CFA. Colonies were fixed and stained with crystal violet after 11 days and their sizes were quantified. We identified a significant reduction in the average size and number of colonies in HeLa-TIP60-pBABE as compared to HeLa-LPCX-pBABE (vector), consistent with our earlier findings [8]. Overexpression of *TERT* in the HeLa-LPCX background did not affect the colony formation ability. Interestingly, overexpression of *TERT* in HeLa-TIP60 cells showed a significant increase in the average size and number of colonies as compared to HeLa-TIP60-pBABE, thus rescuing the growth defect caused by TIP60 overexpression **(Fig 5A, 5B and 5C)**.

### TIP60-dependent acetylation of Sp1 is involved in mediating the growth inhibition in HeLa cells

**Fig 6:**
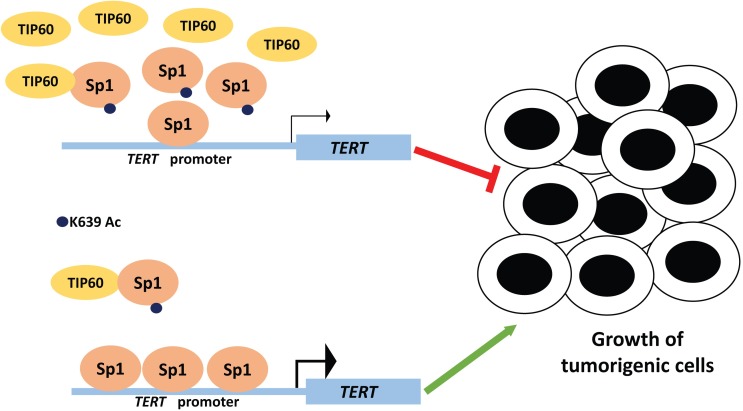
Model of TIP60-mediated *TERT* repression. TIP60 inhibits expression of *TERT* by acetylating Sp1. Decrease in TIP60 levels increases occupancy of Sp1 on *TERT* promoter resulting in increased expression of *TERT* and subsequently tumor growth.

Given that Sp1 acetylation could explain TIP60-dependent changes in *TERT* mRNA levels, we reasoned that this might also be the mechanistic link to explain TIP60-dependent inhibition in colony formation that can be rescued by exogenous expression of *TERT*. To address this, we examined *TERT* mRNA levels and colony formation ability upon expression of the Sp1 wild-type and mutant constructs in HeLa (**S8C Fig**). Overexpression of WT Sp1 as well as Sp1 K639R caused an increase in *TERT* expression, whereas overexpression of Sp1 K639Q mutant did not result in any change in the *TERT* expression (**S8D Fig**). Importantly, overexpression of Sp1 WT and Sp1 K639R resulted in more colonies in CFA in HeLa cells whereas there was no significant difference in the colony formation ability of Sp1 K639Q (**Fig 5D and 5E**). This data underscores the identified regulatory hierarchy in which TIP60 acetylates Sp1 at K639, which inhibits *TERT* mRNA expression and in return impacts on the proliferative potential of the affected cancer cells (**Fig 6**).

## Discussion

Telomerase has been an attractive therapeutic target for cancer therapy because it is selectively activated in cancers and stem cells but not expressed in most differentiated tissues [35]. Although multiple factors regulating *TERT* have been identified in the past, there is a dearth of knowledge regarding mechanisms that repress the endogenous *TERT* levels in normal somatic cells. Human papillomavirus protein, E2, which is known to repress expression of HPV E6 and E7 and is inactivated upon integration of HPV genome into the host, is also known to repress the *TERT* promoter and associated telomerase activity. Furthermore, this repressive function of E2 is mediated by its recruitment to the *TERT* promoter and through Sp1 binding sites [9, 36]. This supports our findings on the significance of Sp1 in regulating *TERT* expression and that negative regulators of Sp1 function are potent repressors of telomerase expression. Tumor suppressors have been shown to negatively regulate telomerase expression [37]and our study identifies for the first time, TIP60, to be a negative regulator of *TERT*. In the past, the unconventional repressive activity associated with the histone acetyl transferase, TIP60, has been studied and it is known to repress viral oncogenes like HPV E6, adenovirus E1A as well as some cellular genes [6, 7, 38]. Since the HPV Long control region (LCR), which regulates transcription of the viral oncogenes, is known to harbor binding sites for Sp1 and we have identified TIP60-mediated acetylation to inhibit Sp1 function at the *TERT* promoter, we were curious to study if modulation of TIP60 levels altered Sp1 occupancy at the HPV LCR. We observed no significant increase in enrichment of Sp1 at HPV LCR regions upon TIP60 depletion (**S9 Fig**) as compared to siControl treated cells. On the contrary, Sp1 occupancy was decreased significantly in one region of HPV LCR upon TIP60 depletion. The specificity of Sp1 ChIP was established by decreased occupancy upon both Sp1 knockdown as well as co-depletion of TIP60 and Sp1 (**S9 Fig**). This hints at the role of perhaps other members of the Sp family of transcription factors or other transcription factors at the HPV LCR region.

Although mechanisms of post-translational and post-transcriptional control of *TERT* have been elucidated, the predominant form of regulation across many cancer types is the transcriptional control [39, 40]. Previous studies have shown a good correlation of *TERT* expression with the promoter activity using luciferase reporter assays [28, 40] thus reiterating the importance of transcriptional control of *TERT* and hence telomerase activity. In our study, HeLa cells overexpressing TIP60 showed a decreased telomerase activity (**Fig 1A**) that is consistent with the decrease in *TERT* expression (**Fig 1C**) and depletion of TIP60 increases telomerase activity (**Fig 1D**) as well as *TERT* expression (**Fig 1F**).

This data indicates that TIP60 represses *TERT* expression in HeLa cells that constitutively express the viral oncoprotein E6, which, together with cellular transcription factors like c-MYC and Sp1 up-regulates *TERT* expression [24, 41] and destabilizes endogenous *TERT* repressors like NFX1-91 to activate the promoter [25]. Our data also shows the dependence on E6 for *TERT* regulation by TIP60 (**S2 Fig**), indicating that this mechanism could be a feature unique to virus-induced tumors. Several other onco-viruses like HBV, Epstein Barr Virus (EBV), Human T-lymphotropic virus 1 (HTLV-1) and adenovirus are also known to up-regulate *TERT* transcriptionally [21, 42, 43]. It is interesting to note that some of these are also known to destabilize TIP60 in a fashion similar to HPV E6 and adenovirus [6, 7]. Thus, we speculate that destabilization of TIP60 by viral onco-proteins, which in turn leads to increased telomerase activity, could be a general mechanism that allows virus infected cells to evade senescence. It will be interesting to validate the results in other virus-induced cancers since this would provide an opportunity for therapeutic intervention specific only to the tumor cells. This idea is further supported by the notion that many viral oncogenes also rely on Sp1 to regulate TERT transcriptionally [20, 44]. Our study has identified a novel role of TIP60 in regulating the function of Sp1 at the *TERT* promoter by acetylating Sp1 at K639 (**Fig 4)** and we speculate that this may be a feature common to all virus-induced cancers.

Sp1 plays an important regulatory role in TERT activation, however, it has been shown that levels of Sp1 binding to the *TERT* promoter region are similar in TERT expressing and negative cells [45], which suggests that there must be a differential regulation of Sp1 activity in tumorigenic cells expressing TERT. From our data, TIP60 seems to be a likely candidate that could functionally regulate Sp1 (**Fig 4**). TIP60-dependent acetylation of Sp1 provides a window for therapeutic intervention in cancers driven by Sp1 overexpression. Identification of the lysine 639 on Sp1 as a specific target of TIP60 offers the possibility of a diagnostic tool for TERT-driven cancers. Earlier reports on post-translational modifications of Sp1 indicate that the acetylation of Sp1 occurs at its DNA binding domain, and that acetylated Sp1 loses its DNA binding ability at different promoters such *p21* and *BAK* [33, 46]. Identification of TIP60 as the acetyltransferase responsible for acetylating Sp1 **(Fig 4C)** leads us to speculate if expression of other downstream targets of Sp1 also changes upon modulating TIP60 levels. Sp1 is also known to play an anti-senescence role in the context of telomere uncapping induced senescence. In cell culture models that use a TRF2 dominant negative mutant, Sp1 expression is down-regulated, leading to senescence phenotype [47], thereby highlighting the importance of this transcription factor in telomere biology and reiterating the possibility to target it in cancers that activate telomerase.

In conclusion, this study identifies a novel transcriptional repressor of the catalytic subunit of telomerase and suggests a mechanism of action mediated at the *TERT* promoter through Sp1 (**Fig 6**). TIP60-mediated *TERT* repression might be one of the mechanisms by which virus-infected cells escape replicative senescence.

## Materials and methods

### Cell culture

HeLa cells (ATCC CCL-2), 293T cells (ATCC CRL-3216) and C-33A cells (ATCC HTB-31) were cultured in Dulbecco’s modified Eagle’s media (DMEM) high glucose (Sigma Cat. No. D-5796) with 10% fetal bovine serum (Sigma Cat. No. F-7524), 1% penicillin streptomycin (Gibco Cat. No. 15140–122), Human keratinocytes (ATCC PCS-200-011) were grown in Dermal Cell Basal Medium (ATCC PCS-200-030) supplemented with Bovine Pituitary Extract (BPE), rh TGF-α, L-glutamine, hydrocortisone hemisuccinate, insulin, epinephrine and apo-transferrin at 37°C and 5% CO_2_. HPV18-transformed keratinocytes (HK18) were generated and grown as described elsewhere [48]. For SILAC labeling, HEK293T cells were incubated in DMEM (-Arg, -Lys) medium containing 10% dialyzed fetal bovine serum (Gibco) supplemented with 42 mg/l ^13^C_6_^15^N_4_ L-arginine and 73 mg/l ^13^C_6_^15^N_2_ L-lysine (Cambridge Isotope Laboratories) or the corresponding non-labeled amino acids, respectively. Stable cell lines used in the study, HeLa-LPCX, HeLa-TIP60 and CaSki-TIP60 were generated as described elsewhere [8].

### siRNA transfection

Sp1 and E6 were depleted by transiently transfecting siRNA targeting the open reading frame of these proteins [2, 7, 32]. A commercially available siRNA for TIP60 (siTIP60) was purchased (Sigma Aldrich, SASI_Hs01_00073301) and used in experiments. Sequence of siTIP60A used was as follows- (Sense 5’ UGAUCGAGUUCAGCUAUGA 3’; antisense 5’ UCAUAGCUGAACUCGAUCA 3’). siSP1B siRNA was purchased from SantaCruz (Cat. No. sc-29487). Briefly, 1 million cells were seeded into a 10-cm plate and 20 nM siRNA (siSp1A, siSP1 B, siTIP60A) or 10 nM siRNA (siTIP60, siE6) was used for transfection along with 15 μl Lipofectamine RNAiMax (Invitrogen, Cat. No. 56532) following the manufacturer’s protocol. Six hours post transfection, the transfection mixture was replaced by growth medium and the cells were harvested 72 h after transfection for further analysis.

### Quantitative telomerase repeat amplification protocol (qTRAP)

qTRAP is a real-time PCR based technique that measures the ability of telomerase to add telomeric repeat sequences to the ends of its substrates. We modified the conventional TRAP assay using the TRAPeze telomerase detection kit (Millipore, Cat. No. S7700) to facilitate accurate detection. Briefly, TIP60 was depleted by siRNA transfection and the cells were harvested after 72 h. The cell pellet was lysed using 1 CHAPS lysis buffer provided with the kit (10 mM Tris-HCl, pH 7.5; 1 mM MgCl_2_; 1 mM EGTA; 0.1 mM benzamidine; 5 mM β-mercaptoethanol; 0.5% CHAPS; 10% glycerol), protein was quantified and then the TRAP reaction was set up with 200 nM of telomerase primer TS (5’ AATCCGTCGAGCAGAGTT 3’) and anchored return primer ACX (5’ GCGCGGCTTACCCTTACCTTACCCTAACC 3’). The reaction was carried out in the ABI Prism 7500 (Applied Biosystems) thermal cycler with the samples incubated for 20 min at 25°C and amplified in 32 PCR cycles with 30 sec at 95°C and 90 sec at 60°C followed by 72°C for 1 min. The threshold C_t_ values were determined and data was analyzed by comparing to a standard curve generated by serial dilution of the siControl/LPCX vector samples. Heat inactivated lysates serve as negative control.

### RNA isolation

Total RNA was isolated using TRIZOL reagent (Life Technologies, Cat. No. 15596–026) according to manufacturer’s protocol.

### Real-time PCR (RT-PCR) analysis

The total RNA was converted into complementary DNA (cDNA) using iSCRIPT cDNA synthesis kit (Bio-Rad Cat. No. 170–8891). This served as a template for RT-PCR analysis using iTaq Universal SYBR Green Supermix (Bio-Rad Cat. No. 172-5124) on an Applied Biosystems 7500 Fast Real Time PCR system using the following conditions for the run—50°C for 2 min, 95°C for 10 min and amplified by 40 cycles of PCR, 95°C for 15 sec and 60°C for 1 min. The melt curves for all qPCR reactions were also obtained. Results were analyzed and represented as fold change. The primers used are specified in the primer list (**Table 1**).

**Table 1:** Quantitative PCR primers.

Gene	Forward primer (5’-3’)	Reverse primer (5’-3’)
*TIP60*	AATGTGGCCTGCATCCTAAC	TGTTTTCCCTTCCACTTTGG
*E6*	CCAGAAACCGTTGAATCCAG	GTTGGAGTCGTTCCTGTCGT
*TERT*	CTACTCCTCAGGCGACAAGG	TGGAACCCAGAAAGATGGTC
*Sp1*	CTATAGCAAAATGCCCCAGGT	TCCACCTGCTGTGTCATCAT

### Western blot analysis

Proteins were separated on 8% SDS-PAGE gel, transferred onto a nitrocellulose membrane, and detected with primary antibody (in 3% milk in TBST or 4% PBST) against α-Actinin (B-12, SantaCruz, Cat. No. sc-166524, 1:1000), E6 (SantaCruz, Cat. No. sc-365089, 1:500), FLAG (SantaCruz, Cat. No. sc-807, 1:1000), Sp1 (SantaCruz, Cat. No. sc-59, 1:500), TIP60 (generated in the lab 1:500), Pan-acetyl lysine (SantaCruz, Cat. No. sc-8663R, 1:500), GFP-B2 (SantaCruz, Cat. No. sc-9996, 1:1000) and GFP (ROCHE, no. 11814460001 1:4000). Quantification of blots was done using Image J software. For the immunoprecipitation experiments, 4 μg of cell lysate from HeLa cells was used and immunoprecipitated using endogenous TIP60 antibody overnight. The interacting Sp1 was detected using the endogenous antibody described earlier by western blotting.

### Transient transfection and luciferase assay

For the luciferase assay, 500 ng of pGL3, pGL3-949 and pGL3-385 were transfected along with 50 ng of Renilla luciferase construct into HeLa-LPCX and HeLa-TIP60 stable cells in a 12-well plate (1×10^5^ cells per well) using 1 μl of Lipofectamine 2000 (Invitrogen Cat. No. 11668019) for each well following the manufacturer’s instructions. The cells were harvested 24 h after transfection and whole cell lysates were used to determine luciferase activity using the Dual-luciferase reporter 1000 assay system (Promega Cat. No. E1980). All firefly luciferase readings were normalized with Renilla luciferase readings.

### Mutagenesis

To generate mutants of Sp1 binding sites on the *TERT* promoter, the QuikChange II XL Site-Directed Mutagenesis Kit (Agilent, Cat. No. 200522) was used according to the manufacturer’s instructions. The primers used for mutagenesis are provided in **Table 2**.

**Table 2:** Site-directed mutagenesis primers.

	Forward primer (5'-3')	Reverse primer (5'-3')	WT sequence (5’-3’)
Sp1mut1	GCCGCGAGGAGAGGTATGGGCCGCGGAAAGG	CCTTTCCGCGGCCCATACCTCTCCTCGCGGC	TCCGCGGCCCCGCCCTCTCCT
Sp1mut2	AGGGGAGGGTTTGGGAGGGCCCGGAGGG	CCCTCCGGGCCCTCCCAAACCCTCCCCT	CCTCCCAGCCCCTCCCCTTC
Sp1mut3	CCCGGAGGGGTTTGGGCCGGGGACCC	GGGTCCCCGGCCCAAACCCCTCCGGG	CGGGTCCCCGGCCCAGCCCCC
Sp1mut4	GGGTCGGGACGGGTATGGGTCCGCGCGGA	TCCGCGCGGACCCATACCCGTCCCGACCC	CCTCCGCGCGGACCCCGCCCCG
Sp1mut5	CGCGCGGAGGAGGTTGAGCTGGAAGGTG	CACCTTCCAGCTCAACCTCCTCCGCGCG	CCCTTCACCTTCCAGCTCCGCCTCCT
Myc mutant	CAGTCCCTCCGCCACAAAGGAAGCGCGGTCCTG	CAGGACCGCGCTTCCTTTGTGGCGGAGGGACTG	ACCGCGCTTCCCACGTGGC

### Chromatin immunoprecipitation (ChIP)

ChIP was performed following the protocol as described elsewhere (7). Briefly, cells were cross-linked with 1% formaldehyde for 10 min at room temperature. The cells were lysed using SDS Lysis buffer for ChIP (1% SDS, 10 mM EDTA, 50 mM Tris-HCl pH 8). The lysate was then sonicated for 15 cycles at 40% amplitude (15 sec off ON and 45 sec OFF). The sonicated samples were then diluted in ChIP dilution buffer (0.01% SDS, 1% Triton X100, 1.2 mM EDTA, 16.7 mM Tris-HCl pH 8, 167 mM NaCl) and used for the immunoprecipitation with anti-Sp1 or anti-GFP antibody. After an over-night incubation with 10 μg of anti-Sp1 antibody or 4 μg of GFP antibody, the bound DNA was washed sequentially with low salt wash buffer (0.1% SDS, 1% Triton X 100, 2 mM EDTA, 20 mM Tris-HCl pH8, 150 mM NaCl), high salt wash buffer (0.1% SDS, 1% Triton X 100, 2 mM EDTA, 20 mM Tris-HCl pH 8, 500 mM NaCl), LiCl wash buffer (0.25 M LiCl, 1% NP40, 1% deoxycholate, 1 mM EDTA, 10 mM Tris-HCl pH 8) and TE wash buffer (10 mM Tris-HCl pH 8, 1 mM EDTA) to remove non-specific sequences and eluted in the elution buffer (84 mg NaHCO_3_, 1 ml 10% SDS, 9 ml H_2_O). Then the samples were reverse cross-linked using NaCl at 65°C for 4 h. The eluted DNA was purified and used for qPCR. qPCR was performed in 40 cycles with 95°C for 15 sec and 53°C for 1 min for *TERT* promoter primers and 95°C for 15 sec and 60°C for 1 min for *TERT* upstream primers with the products of ChIP, using primers described in **Table 3**.

**Table 3:** Chromatin immunoprecipitation primers.

	Forward primer (5'-3')	Reverse primer (5'-3')
*TERT* promoter	TGCCCCTTCACCTTCCA	CTGAAACTCGCGCCG
*TERT* upstream element	GGCTACTGCACGCACCTTTTA	CAAAGATGGCACAGCCTCCG

### GFP pull-down

293T cells were transiently transfected with plasmid expressing EGFP-Sp1 (2 μg) in the presence of TIP60 (2 μg) or a catalytically dead form of TIP60 (2 μg). Cells were harvested 48 h post transfection, after treatment with Histone deacetylase (HDAC) inhibitors (5 μM Sodium butyrate, 1 μM Trichostatin A and 1 μM Nicotinamide) for 5 h prior to harvest. Cells were lysed using lysis buffer (10 mM Tris Cl pH 7.5, 150 mM NaCl, 0.5 M EDTA and 0.2% NP-40) and incubated with GFP-TRAP_A beads (Chromotek, Cat. No. gta-20) following the manufacturer’s protocol. The pull-down samples were analyzed by western blotting and the acetylation on Sp1 was detected using a pan-acetyl lysine antibody.

### Colony formation Assay

2×10^3^ cells were seeded and allowed to grow in complete medium supplemented with antibiotics for 11 days. The colonies were fixed and stained using 0.1% Crystal violet in 20% methanol. The images were quantified using Image J software (http://imagej.nih.gov/ij/). The parameters for particle size on Image J were set from 0-infinity and the circularity as 0–1 and all particles in this dimension were included to calculate number and size of colonies.

### Mass spectrometry analysis

In-gel digestion and MS analysis was performed essentially as previously described by Butter *et al* [49]. In brief, samples were reduced in 10 mM DTT 1 h at 56°C followed by alkylation with 55 mM iodoacetamide (Sigma) for 45 min in the dark. Tryptic digest was performed in 50 mM ammonium bicarbonate buffer with 2 μg trypsin (Promega) at 37°C overnight. Peptides were desalted on StageTips and analyzed by nanoflow liquid chromatography on an EASY-nLC 1200 system coupled to a Q Exactive HF mass spectrometer (Thermo Fisher Scientific). Peptides were separated on a C18-reversed phase column (25 cm long, 75 μm inner diameter) packed in-house with ReproSil-Pur C18-QAQ 1.9 μm resin (Dr Maisch). The column was mounted on an Easy Flex Nano Source and temperature controlled by a column oven (Sonation) at 40°C. A 105-min gradient from 2 to 40% acetonitrile in 0.5% formic acid at a flow of 225 nl/min was used. Spray voltage was set to 2.4 kV. The Q Exactive HF was operated with a TOP20 MS/MS spectra acquisition method per MS full scan. MS scans were conducted with 60,000 and MS/MS scans with 15,000 resolution. The raw files were processed with MaxQuant [50] version 1.5.2.8 with preset standard settings for SILAC-labeled samples digested with trypsin and the re-quantify option was activated. Carbamidomethylation was set as fixed modification while methionine oxidation, protein N-acetylation and lysine-acetylation were considered as variable modifications. Search results were processed with MaxQuant filtered with a false discovery rate of 0.01.

### *In vitro* DNA reconstitution pull-down

Biotinylated DNA baits consisting of the 2^nd^ Sp1 binding site in the *TERT* promoter or a mutated sequence were prepared as previously described [51]. For primer sequences see **Table 4**. Sp1 WT or Sp1 mut DNA was immobilized on 250 μg paramagnetic streptavidin beads (Dynabeads MyOne C1, Life Technologies) on a rotation wheel for 30 min at room temperature. Subsequently, baits were incubated with 100 μg of nuclear extract in PBB buffer (150 mM NaCl, 50 mM Tris-HCl pH 7.5, 5 mM MgCl_2_, 0.5% Igepal CA-630 (Sigma)) while rotating for 2 h at 4°C. 20 μg sheared salmon sperm DNA (Ambion) was added as a competitor for DNA binding. After three washes with PBB buffer, bound proteins were eluted in 2× Laemmli buffer (Sigma-Aldrich), boiled for 5 min at 95°C and separated on a 4–12% NuPAGE Novex Bis-Tris precast gel (Life Technologies).

**Table 4:** DNA pull-down primers.

	Forward primer (5'-3')	Reverse primer (5'-3')
WT Probe	AGAGGAAGGGGAGGGGCTGGGAGGGCCCGG	CTCCGGGCCCTCCCAGCCCCTCCCCTTCCT
Mutant Probe	AGAGGAAGGGGAGGGTTTGGGAGGGCCCGG	CTCCGGGCCCTCCCAAACCCTCCCCTTCCT

### Statistical analysis

All statistical analyses were performed using unpaired two-tailed student’s *t-*test. Error bars indicate standard error of mean (SEM) for the indicated number of biological repeats. Significance is represented as *P<0.05, **P<0.01, ***P<0.001.

## Supporting information

S1 Fig Depletion of TIP60 increases TERT activity in HeLa and CaSki-TIP60 cells. **(A)** TIP60 was transiently depleted in HeLa-TIP60 cells using siTIP60A and cells were harvested 72 h post transfection. Telomerase activity was measured. **(B)** Efficiency of TIP60 depletion was confirmed by western blotting. TIP60 was detected using an endogenous TIP60 antibody and Actin serves as loading control. **(C)** Cellular RNA was isolated and analyzed by real time PCR after knockdown to determine *TERT* expression. mRNA expression was normalized to *GAPDH* and plotted as fold change. (**D**) Transient depletion of TIP60 in HeLa cells using siRNA transfection and measurement of telomerase activity by qTRAP. **(E)** Western blotting analysis on the same set of cells to verify the knockdown of TIP60. Endogenous TIP60 was detected using anti-TIP60 antibody. **(F)** Real-time PCR analysis to study the expression of *TERT* upon TIP60 knockdown. mRNA data was normalized to *GAPDH* and plotted as fold change. (**G**) Relative telomerase activity was measured after transient knockdown of TIP60 using siTIP60 in CaSki-TIP60 cells. (**H**) Depletion of TIP60 was verified by western blotting analysis using anti-TIP60 antibody to detect TIP60. All the bands observed when probed with anti-TIP60 antibody correspond to TIP60 protein since they are specifically reduced upon depletion of TIP60. (**I**) *TERT* expression was also checked after TIP60 depletion using qPCR. mRNA data was normalized to *GAPDH* and plotted as fold change. Error bars reflect the standard error of mean (SEM) of 3 independent experiments and significance is represented as *, *P*<0.05, ***, *P*<0.001.
(TIF)

S2 Fig Mechanistic investigation of involvement of HPV E6 in TIP60-mediated *TERT* repression. (**A**) Co-depletion of TIP60 and E6 using siRNA was performed in HeLa cells. Seventy two hours post transfection of TIP60 and E6 siRNA, cells were harvested and analyzed by western blotting with antibodies to detect endogenous TIP60 and E6. All the bands observed when probed with anti-TIP60 antibody correspond to TIP60 protein since they are specifically reduced upon depletion of TIP60. α-Actinin serves as an internal control as well as to indicate equal protein amount used for qTRAP assay. (**B**) Telomerase activity was measured for the same set of samples using qTRAP. (**C**) C-33A cells are cervical cancer cells which are not infected by HPV. In these cells, TIP60 was transiently depleted using siRNA and cells were harvested 72 h post transfection. Real-time PCR analysis was used to study the expression of *TERT* upon TIP60 depletion. Error bars reflect the standard error of mean (SEM) of 3 independent experiments and significance is represented as *, *P*<0.05, **, *P*<0.01
(TIF)

S3 Fig Depletion of TIP60 in HeLa-TIP60 cells results in *TERT* induction. **(A, B)** Transient depletion of TIP60 using siRNA and increase in *TERT* expression was verified in the same set of cells used for luciferase assay in **Fig 2B**.
(TIF)

S4 Fig TIP60-mediated *TERT* repression is dependent on Sp1. **(A, B)** TIP60 and Sp1 were depleted in HeLa cells using siRNA transfection. Sp1 was depleted using a second siRNA, siSp1B. Cells were harvested 72 h post transfection, RNA and protein was isolated to use for real time PCR and western blotting analysis. All mRNA expression data was normalized to *GAPDH* and plotted as fold change. Endogenous TIP60 and Sp1 were detected using anti-TIP60 and anti-Sp1 antibodies respectively.
(TIF)

S5 Fig MYC and MAZ are not involved in TIP60 mediated *TERT* repression. (**A, B**) Representation of MYC binding site and the mutant generated on the *TERT* promoter (385 construct) in the pGL3 vector. (**C**) Luciferase reporter gene assay with the MYC binding site mutation. Relative luciferase activity was calculated as a ratio of the firefly luciferase signal to the Renilla luciferase signal. (**D**) Diagrammatic representation of the *TERT* promoter region to describe the location of primers designed for ChIP. The *TERT* promoter primers amplify a region 200 bp upstream of the transcription start site (TSS). The upstream element primers amplify a region of the promoter 3500 bp upstream of the TSS. (**E**) Transient co-depletion of TIP60 and MAZ in HeLa cells and detection of *TERT* expression by qPCR.
(TIF)

S6 Fig TIP60 interacts with Sp1 and acetylates it within the DNA binding domain. (**A**) Endogenous TIP60 protein was pulled down from HeLa cell lysates and associated Sp1 protein was detected by endogenous Sp1 antibody, both in the presence and absence of DNAse. (**B**) Schematic representation of the steps in the SILAC based mass spectrometry analysis. (**C**) Quantification of the incorporation rate of ‘heavy’ arginine and lysine in the cells used for the SILAC experiment. (**D**) Representation of the different domains of Sp1 including the three Zinc finger motifs as obtained from UniProt (http://www.uniprot.org/).
(TIF)

S7 Fig Representative spectra of acetylated Sp1 peptides. (A) Representative MS1 spectra of the Sp1 peptide VYGK(ac)TSHLR^2+^. In the forward experiment (upper panel) the heavy peptide is more abundant than the light peptide, whereas in the reverse experiment (lower panel) the opposite trend is visible. (B) Representative MS2 spectra for the VYGK(ac)TSHLR^2+^ peptide both with heavy (upper panel) and light labeling (lower panel) providing sequence evidence for the existence of the Sp1 modification. (C) Representative MS2 spectra providing sequence evidence for 4 additional Sp1 acetylation sites that according to the SILAC quantification are not dependent on TIP60.
(TIF)

S8 Fig Overexpression of *TERT* and Sp1 in HeLa cells. **(A, B)** mRNA expression data of *TERT* normalized to *GAPDH* and plotted as fold change from cells seeded for CFA in Fig 5A. (**C, D**) Sp1 constructs were transiently transfected into HeLa cells and 24 h post transfection, cells were seeded for CFA. The remaining cells were used to isolate RNA and study *Sp1* and *TERT* expression respectively, by qPCR.
(TIF)

S9 Fig TIP60 depletion does not increase Sp1 occupancy at the HPV LCR. **(A)** Graphical representation of the primers designed along HPV LCR. **(B)** Sp1 ChIP was performed in HeLa cells transiently depleted of either TIP60 or Sp1 alone or together. Three regions along the HPV LCR were used to detect Sp1 occupancy upon modulating TIP60 levels. Error bars represent standard error of mean (SEM) of three independent experiments.
(TIF)

S1 Table Protein identified in the mass spectrometry. List of all the peptides identified in our experimental set up.
(XLSX)
