# Supplementary figures and images for: TIP60 represses telomerase expression by inhibiting Sp1 binding to the TERT promoter
Source: PLoS Pathog. 2017 Oct 18;13(10):e1006681. doi: 10.1371/journal.ppat.1006681 (PMC5662243; doi:10.1371/journal.ppat.1006681)

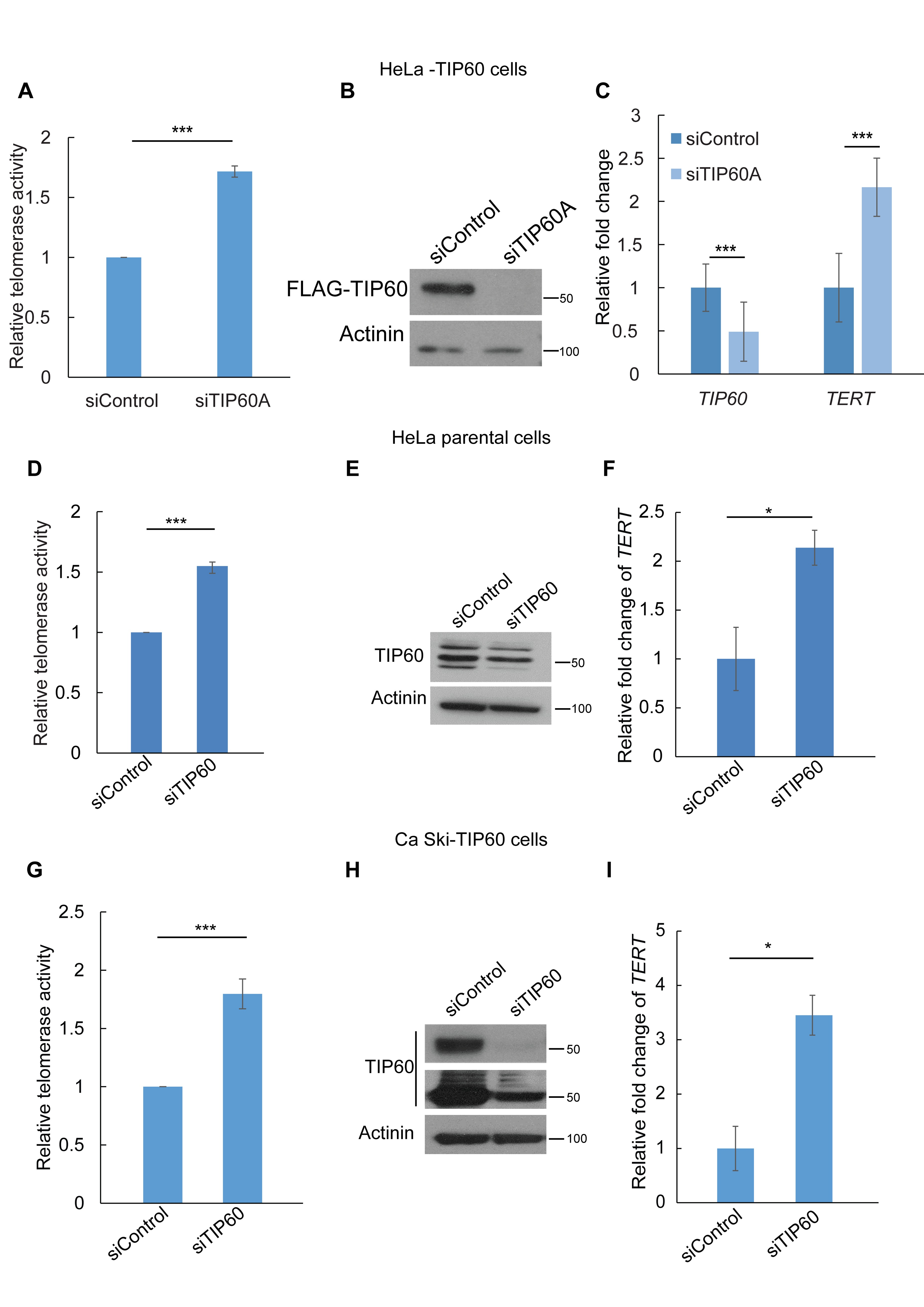

Supplement: S1 Fig — (A) TIP60 was transiently depleted in HeLa-TIP60 cells using siTIP60A and cells were harvested 72 h post transfection. Telomerase activity was measured. (B) Efficiency of TIP60 depletion was confirmed by western blotting. TIP60 was detected using an endogenous TIP60 antibody and Actin serves as loading control. (C) Cellular RNA was isolated and analyzed by real time PCR after knockdown to determine TERT expression. mRNA expression was normalized to GAPDH and plotted as fold change. (D) Transient depletion of TIP60 in HeLa cells using siRNA transfection and measurement of telomerase activity by qTRAP. (E) Western blotting analysis on the same set of cells to verify the knockdown of TIP60. Endogenous TIP60 was detected using anti-TIP60 antibody. (F) Real-time PCR analysis to study the expression of TERT upon TIP60 knockdown. mRNA data was normalized to GAPDH and plotted as fold change. (G) Relative telomerase activity was measured after transient knockdown of TIP60 using siTIP60 in CaSki-TIP60 cells. (H) Depletion of TIP60 was verified by western blotting analysis using anti-TIP60 antibody to detect TIP60. All the bands observed when probed with anti-TIP60 antibody correspond to TIP60 protein since they are specifically reduced upon depletion of TIP60. (I) TERT expression was also checked after TIP60 depletion using qPCR. mRNA data was normalized to GAPDH and plotted as fold change. Error bars reflect the standard error of mean (SEM) of 3 independent experiments and significance is represented as *, P<0.05, ***, P<0.001. (TIF) [file ppat.1006681.s001.tif]

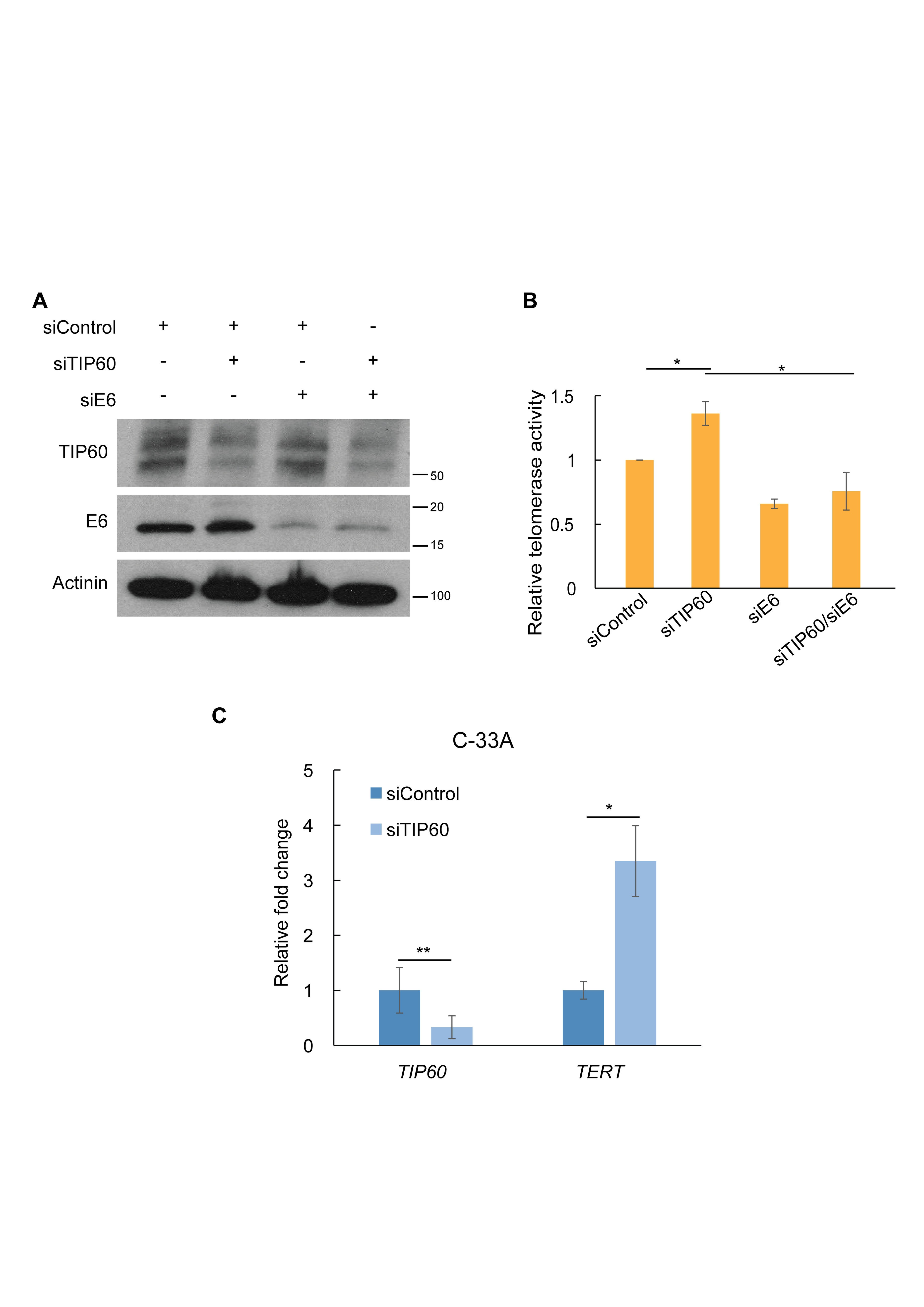

Supplement: S2 Fig — (A) Co-depletion of TIP60 and E6 using siRNA was performed in HeLa cells. Seventy two hours post transfection of TIP60 and E6 siRNA, cells were harvested and analyzed by western blotting with antibodies to detect endogenous TIP60 and E6. All the bands observed when probed with anti-TIP60 antibody correspond to TIP60 protein since they are specifically reduced upon depletion of TIP60. α-Actinin serves as an internal control as well as to indicate equal protein amount used for qTRAP assay. (B) Telomerase activity was measured for the same set of samples using qTRAP. (C) C-33A cells are cervical cancer cells which are not infected by HPV. In these cells, TIP60 was transiently depleted using siRNA and cells were harvested 72 h post transfection. Real-time PCR analysis was used to study the expression of TERT upon TIP60 depletion. Error bars reflect the standard error of mean (SEM) of 3 independent experiments and significance is represented as *, P<0.05, **, P<0.01 (TIF) [file ppat.1006681.s002.tif]

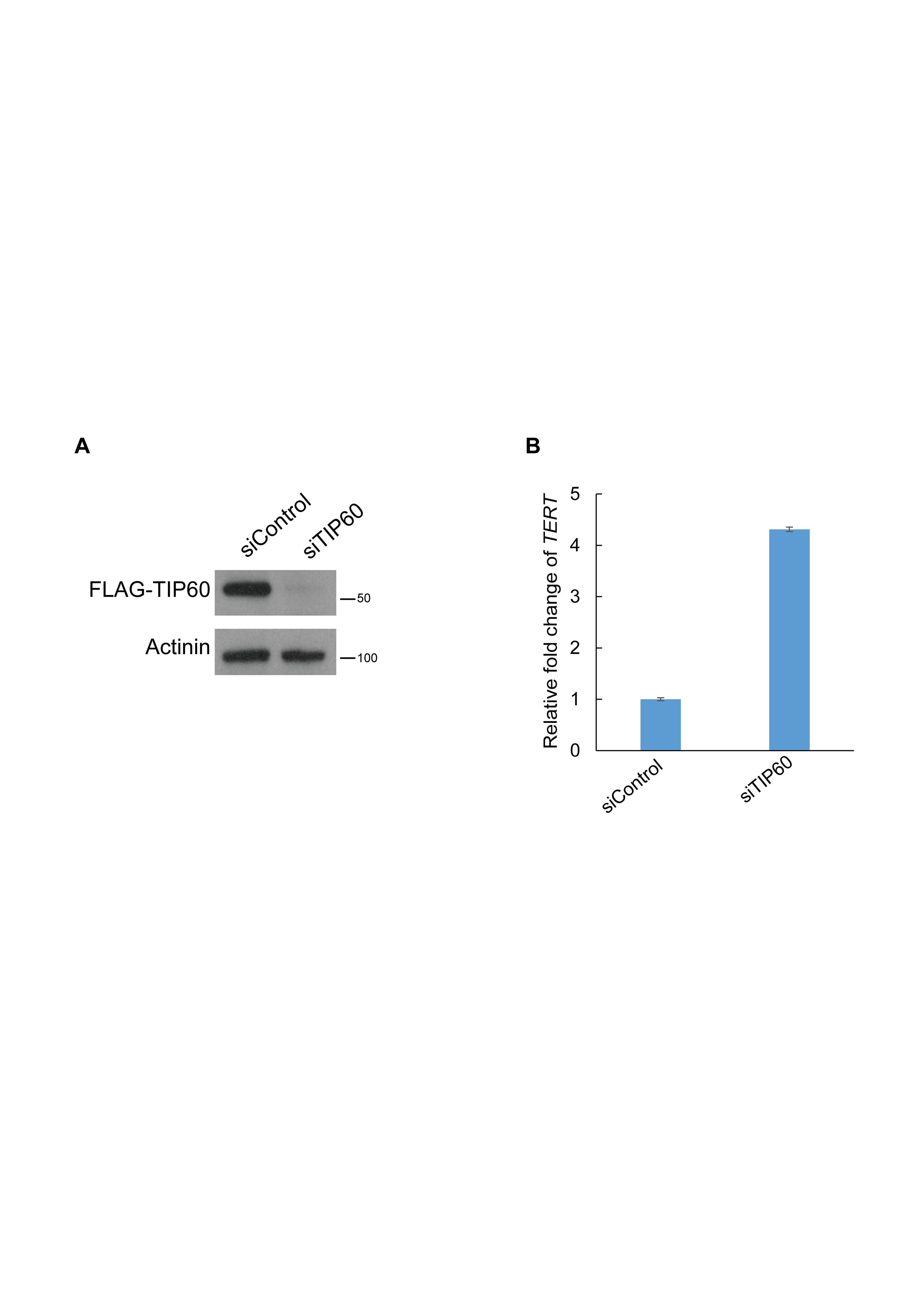

Supplement: S3 Fig — (A, B) Transient depletion of TIP60 using siRNA and increase in TERT expression was verified in the same set of cells used for luciferase assay in Fig 2B. (TIF) [file ppat.1006681.s003.tif]

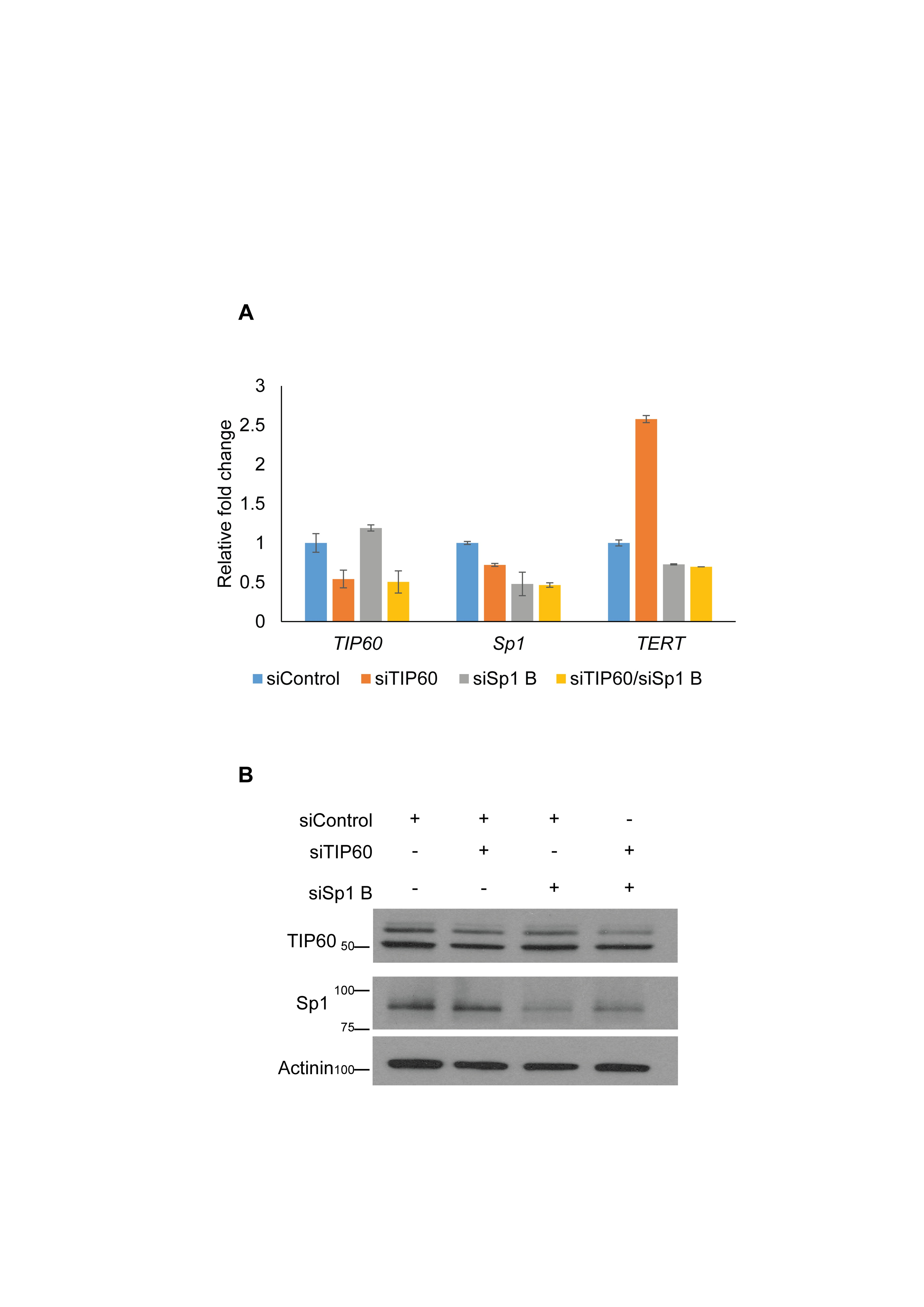

Supplement: S4 Fig — (A, B) TIP60 and Sp1 were depleted in HeLa cells using siRNA transfection. Sp1 was depleted using a second siRNA, siSp1B. Cells were harvested 72 h post transfection, RNA and protein was isolated to use for real time PCR and western blotting analysis. All mRNA expression data was normalized to GAPDH and plotted as fold change. Endogenous TIP60 and Sp1 were detected using anti-TIP60 and anti-Sp1 antibodies respectively. (TIF) [file ppat.1006681.s004.tif]

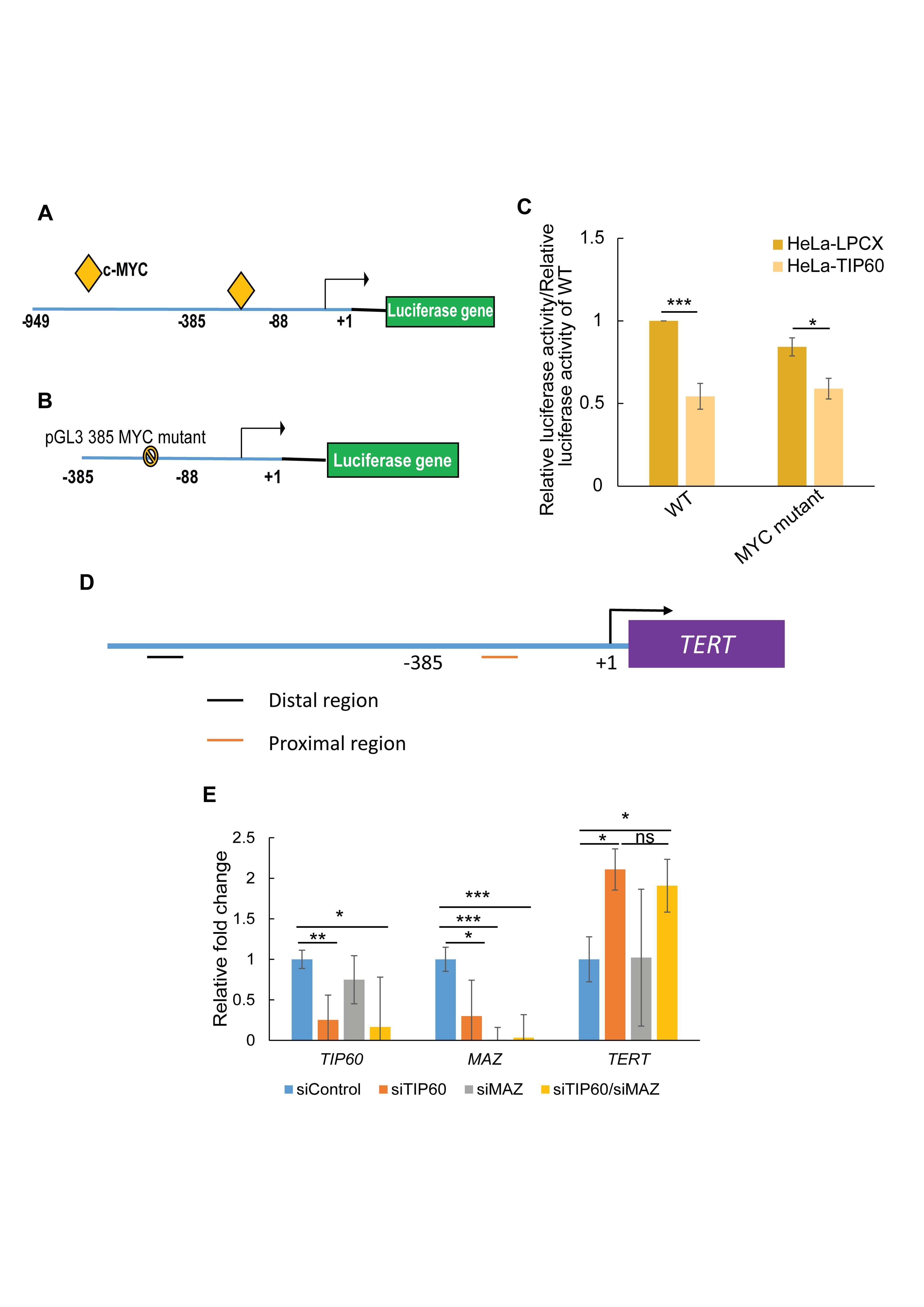

Supplement: S5 Fig — (A, B) Representation of MYC binding site and the mutant generated on the TERT promoter (385 construct) in the pGL3 vector. (C) Luciferase reporter gene assay with the MYC binding site mutation. Relative luciferase activity was calculated as a ratio of the firefly luciferase signal to the Renilla luciferase signal. (D) Diagrammatic representation of the TERT promoter region to describe the location of primers designed for ChIP. The TERT promoter primers amplify a region 200 bp upstream of the transcription start site (TSS). The upstream element primers amplify a region of the promoter 3500 bp upstream of the TSS. (E) Transient co-depletion of TIP60 and MAZ in HeLa cells and detection of TERT expression by qPCR. (TIF) [file ppat.1006681.s005.tif]

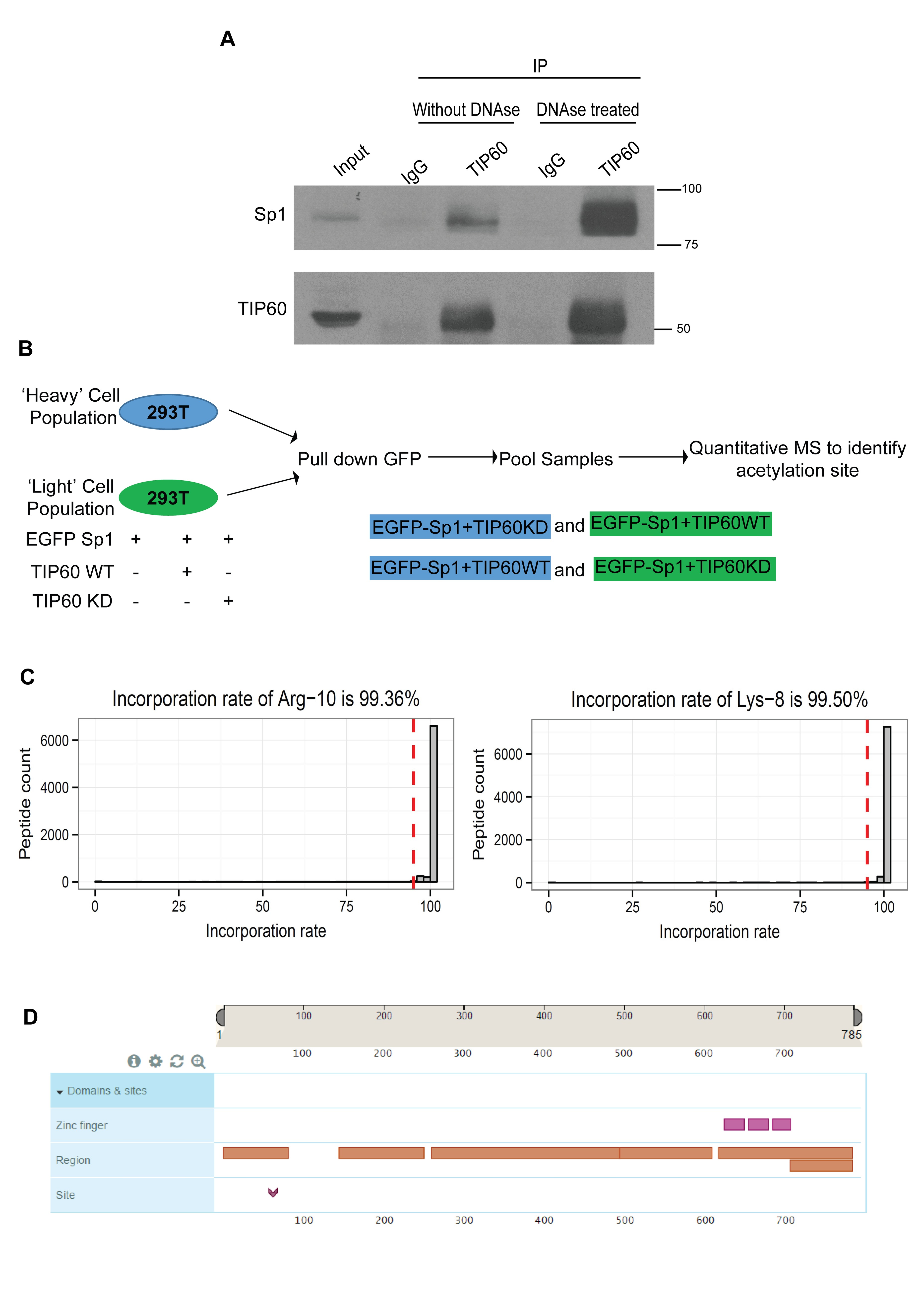

Supplement: S6 Fig — (A) Endogenous TIP60 protein was pulled down from HeLa cell lysates and associated Sp1 protein was detected by endogenous Sp1 antibody, both in the presence and absence of DNAse. (B) Schematic representation of the steps in the SILAC based mass spectrometry analysis. (C) Quantification of the incorporation rate of ‘heavy’ arginine and lysine in the cells used for the SILAC experiment. (D) Representation of the different domains of Sp1 including the three Zinc finger motifs as obtained from UniProt (http://www.uniprot.org/). (TIF) [file ppat.1006681.s006.tif]

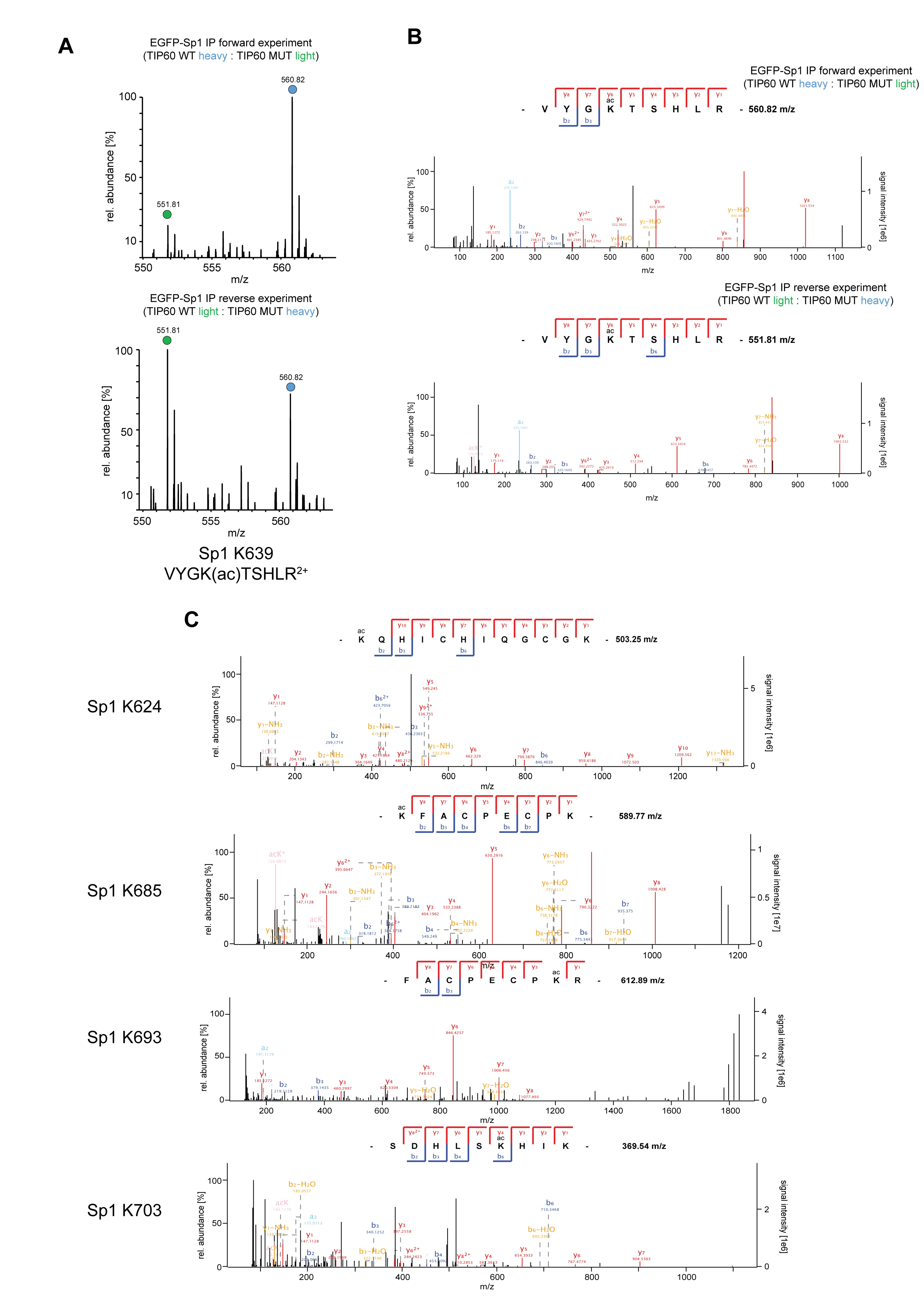

Supplement: S7 Fig — (A) Representative MS1 spectra of the Sp1 peptide VYGK(ac)TSHLR2+. In the forward experiment (upper panel) the heavy peptide is more abundant than the light peptide, whereas in the reverse experiment (lower panel) the opposite trend is visible. (B) Representative MS2 spectra for the VYGK(ac)TSHLR2+ peptide both with heavy (upper panel) and light labeling (lower panel) providing sequence evidence for the existence of the Sp1 modification. (C) Representative MS2 spectra providing sequence evidence for 4 additional Sp1 acetylation sites that according to the SILAC quantification are not dependent on TIP60. (TIF) [file ppat.1006681.s007.tif]

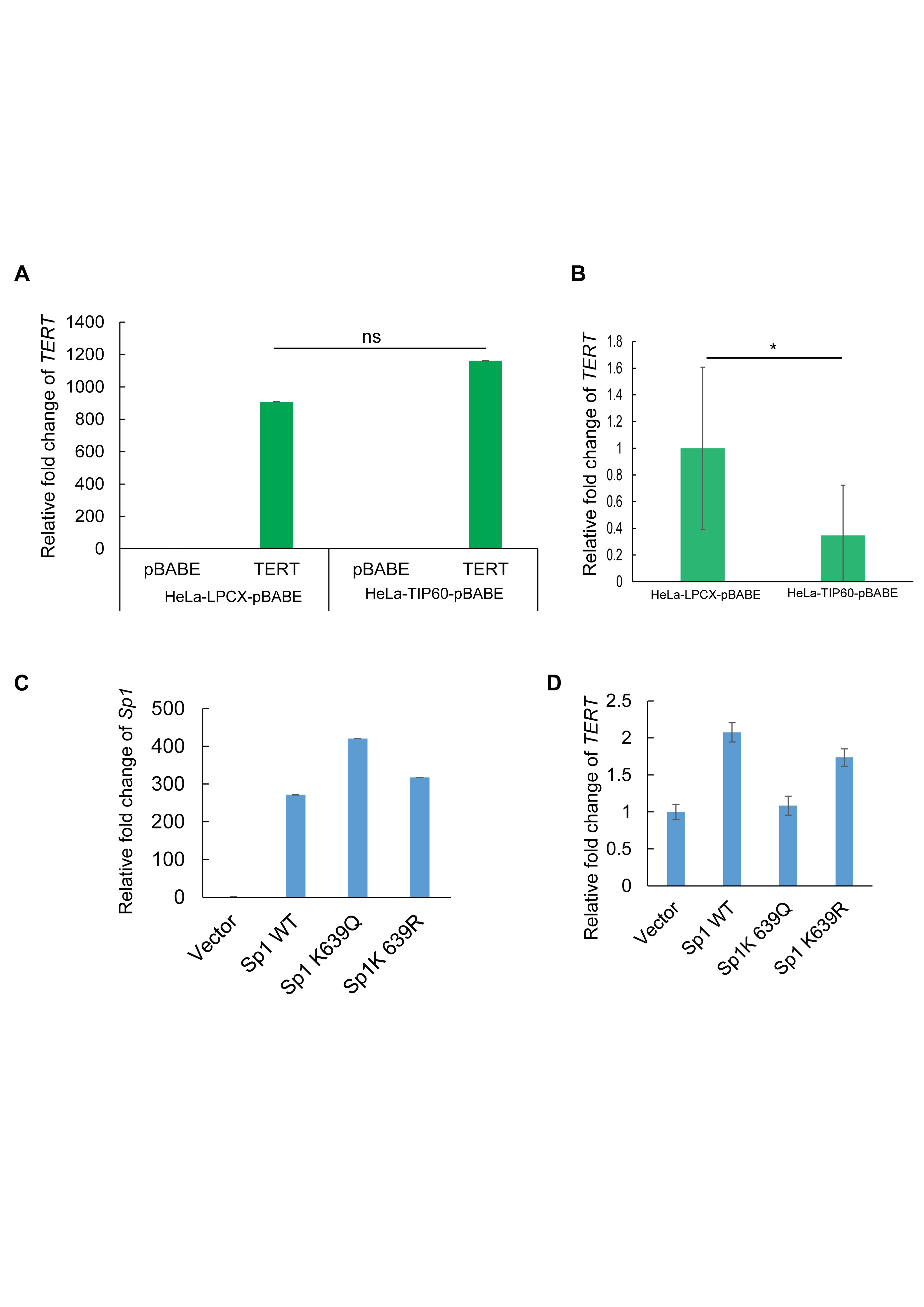

Supplement: S8 Fig — (A, B) mRNA expression data of TERT normalized to GAPDH and plotted as fold change from cells seeded for CFA in Fig 5A. (C, D) Sp1 constructs were transiently transfected into HeLa cells and 24 h post transfection, cells were seeded for CFA. The remaining cells were used to isolate RNA and study Sp1 and TERT expression respectively, by qPCR. (TIF) [file ppat.1006681.s008.tif]

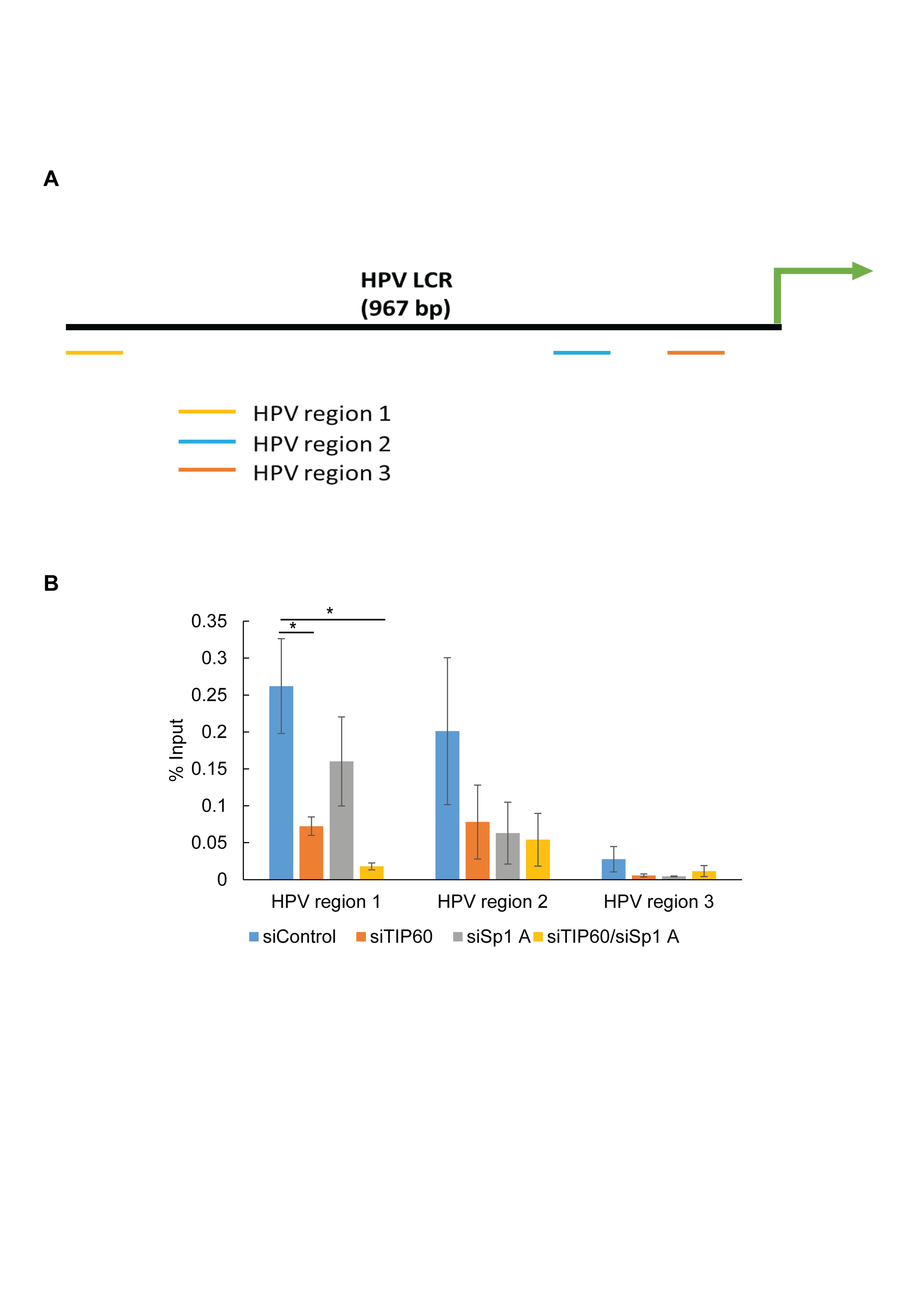

Supplement: S9 Fig — (A) Graphical representation of the primers designed along HPV LCR. (B) Sp1 ChIP was performed in HeLa cells transiently depleted of either TIP60 or Sp1 alone or together. Three regions along the HPV LCR were used to detect Sp1 occupancy upon modulating TIP60 levels. Error bars represent standard error of mean (SEM) of three independent experiments. (TIF) [file ppat.1006681.s009.tif]
